# Effect of Ubiquinol on Cognitive Function, Blood Pressure, Arterial Stiffness, and Biomarkers of Oxidative Stress and Inflammation in the Elderly: A Randomized Trial

**DOI:** 10.3390/antiox15070806

**Published:** 2026-06-27

**Authors:** Madeleine C. Nankivell, Franklin Rosenfeldt, Jeffery M. Reddan, Judy B. de Haan, Cherry Zhang Ping, Beaudan Campbell-Brown, Andrew Pipingas, Matthew P. Pase, Ruchong Ou, Matthew B. Cooke, David L. Hare, Con Stough

**Affiliations:** 1 Centre for Mental Health and Brain Sciences, Swinburne University, Melbourne 3122, Australia; 2 Cardiovascular Inflammation and Redox Biology Lab, Baker Heart & Diabetes Institute, Melbourne 3004, Australia; judy.dehaan@baker.edu.au (J.B.d.H.); ruchong.ou@baker.edu.au (R.O.); 3 School of Psychological Sciences, Turner Institute for Brain and Mental Health, Monash University, Melbourne 3168, Australiamatthew.pase@monash.edu (M.P.P.); 4 Sport, Performance and Nutrition Research Group, School of Allied Health, Human Services and Sport, La Trobe University, Bundoora 3086, Australia; 5 School of Health Science, Swinburne University, Melbourne 3122, Australia; 6 Department of Cardiology, Austin Health, Melbourne 3084, Australia; david.hare@unimelb.edu.au; 7 Faculty of Medicine, Dental and Health Sciences, University of Melbourne, Melbourne 3010, Australia; 8 Metavate Consulting, Sydney 2000, Australia

**Keywords:** CoQ10, ubiquinol, cognition, aging, cardiovascular, oxidative stress, inflammation

## Abstract

Older age is typically characterized by decrements in cognitive performance relative to younger adults, though it may not necessarily reach clinical impairment. Dietary supplementation with ubiquinol, the reduced form of the antioxidant and cellular energizer CoQ10, may support cognitive function in older individuals. In the current randomized clinical trial of 111 adults aged 60 years and older (ubiquinol, n = 61; placebo, n = 50), 90 days of ubiquinol (200 mg) supplementation resulted in plasma CoQ10 levels being four times that of the placebo group at study end (*p* < 0.001). We found that ubiquinol supplementation did not facilitate group differences (controlling for baseline values and relevant demographics) in cognitive function, blood biomarkers reflective of oxidative stress or inflammation, measures of cardiovascular health, or subjective mood at study end. However, regression analyses revealed a positive association between change in plasma CoQ10 and memory performance, as well as a negative association between change in oxidative stress and memory performance at study end in those who received ubiquinol but not placebo. We conclude that adequately powered future clinical trials should examine whether long-term supplementation with ubiquinol can support cognitive function in older adults at risk of cognitive decline or with health conditions predisposing them to risk factors associated with decline.

## 1. Introduction

The proportion of adults aged over 60 is increasing, with 22% of the global population in 2050 predicted to be 60 years or older [1]. This demographic shift has implications for regional and global health systems as older age is a major risk factor for neurodegeneration, with dementia being the most common neurodegenerative syndrome [2]. Importantly, in Australia, dementia is already the leading cause of death [3]. However, cognitive performance normally declines with age and may not always progress to clinically defined cognitive impairment. Moreover, there are very limited pharmacological options for mitigating such decline. Consequently, there is considerable interest in identifying non-pharmacological or lifestyle interventions for supporting healthier cognitive aging.

Various nutritional interventions or supplements have been investigated regarding their efficacy for supporting cognitive function in older age. Two pertinent examples being the ‘Mediterranean diet’ (MeDi) and omega-3 polyunsaturated fatty acid (OM-3 PUFA) supplementation, both of which have been shown to benefit cognitive function in older adults [4,5,6,7]. Greater MeDi adherence or OM-3 PUFA intake also appear to facilitate reduced oxidative stress [8,9,10] and inflammation [8,11,12] as well as healthier blood pressure [13,14,15] and reduced arterial stiffness [16,17]. Considering that elevated oxidative stress [18,19], inflammation [18,20], elevated blood pressure [21,22] as well as increased arterial stiffness [23] have been shown to predict poorer cognitive performance, their amelioration likely contributes to the cognitive benefits associated with greater MeDi adherence and OM-3 PUFA intake.

Another therapeutic intervention that may benefit cognitive function, and which may represent a simpler and potentially more accessible strategy for older adults who experience difficulty adhering to long-term dietary modification, is supplementation with co-enzyme Q10 (CoQ10), particularly in its reduced form, ubiquinol. CoQ10 is a naturally occurring fat-soluble antioxidant and cellular energizer found in all cells of the body, being especially concentrated in mitochondria [24,25]. A recent review by our group [26] identified multiple animal studies (rats and mice) reporting benefits to spatial memory and learning following CoQ10 administration compared to controls. However, the administered doses of CoQ10 in animal studies vary and can often be very high and unlikely to be feasible, or sustainable, in human subjects.

The extent to which CoQ10 supplementation may benefit cognitive function in older adults has received little attention. Indeed, while clinical trials have investigated potential cognitive benefits in older adults, studies have predominantly utilized prodromal (e.g., mild cognitive impairment) and clinical disease groups such as Alzheimer’s dementia, Parkinson’s disease, progressive supranuclear palsy, and chronic fatigue syndrome [26]. As discussed in our earlier review [26], the results of these clinical trials regarding the cognitive benefits of CoQ10 administration is mixed with significant benefits to cognitive performance largely being observed in specific populations, such as Parkinson’s disease [27], progressive supranuclear palsy [28], and chronic fatigue syndrome [29].

To date, only two randomized clinical trials of CoQ10 on cognition in healthy subjects have been published. The first of these was a randomized double-blind controlled trial by Kennedy et al. [30]. This study assessed the impact of a low dose of CoQ10 (4.5 mg per day) in conjunction with multivitamin and mineral supplements in 106 females aged between 25 and 49 years. It was found that when completing cognitive tasks, cerebral blood flow significantly increased in the treatment group but there was no associated change in task performance. In the second study [31], 90 healthy adults aged between 50 and 83 years were given 50 mg ubiquinol capsules twice daily (i.e., 100 mg). After 34 weeks, there were no overall differences in cognition between the treatment and control groups. However, in a subgroup of subjects who were cognitively normal at baseline, ubiquinol facilitated a significant improvement in memory, attention and processing speed.

Considering the relative paucity of trials assessing the effects of CoQ10 on cognition in disease-free older adults, it is clear additional well-designed clinical trials are required.

While exploring potential mechanisms through which CoQ10 could influence cognitive performance was not the focus of our recent review [26], we identified several potential beneficial mechanisms across different animal and human studies. In animal studies, potential mechanisms included reduced presence of histological markers associated with Alzheimer’s disease [32,33], as well as reduced oxidative stress [34] and decreased neuroinflammation [35,36]. Human trials also reported improved cerebral blood flow [30] and cerebral vasoreactivity [37] following CoQ10 supplementation. Additional evidence of benefits to endothelial function has been provided by Sabbatinelli et al. [38] and Kawashima et al. [39]. Specifically, Sabbatinelli et al. [38] determined that ubiquinol significantly ameliorated dyslipidemia-related endothelial dysfunction. This effect was strongly related to increased nitric oxide bioavailability and was partly mediated by enhanced LDL antioxidant protection. Likewise, Kawashima et al. [39] observed that peripheral endothelial function improved within stable heart-failure patients.

Several large meta-analyses of human randomized clinical trials also attest to significant beneficial effects of CoQ10 supplementation on oxidative stress [40,41,42,43], inflammation [43,44,45], and elevated blood pressure [46,47]. There is also evidence that the provision of CoQ10 could improve arterial stiffness, particularly in those with higher initial arterial stiffness [48]. As indicated above, elevations in oxidative stress, inflammation, high blood pressure, and arterial stiffness are predictive of poorer cognitive performance in older age. Thus, in addition to emerging evidence for beneficial cognitive effects, there appear to be several viable, and interconnected, mechanisms by which CoQ10 intervention could potentially benefit cognitive function in older adults.

The current study aimed to determine whether 90 days of supplementation with ubiquinol (CoQ10) would benefit cognitive performance in older adults. Considering results from previous animal and human clinical studies, it was hypothesized that significantly greater cognitive performance would be observed in older adults after 90 days of ubiquinol supplementation compared to those who received a placebo. It was also proposed that the groups receiving 90 days of ubiquinol supplementation would demonstrate lower oxidative stress and inflammation as well as healthier cardiovascular function compared to placebo at the end of the trial. The extent to which ubiquinol supplementation facilitates healthier subjective mood was also assessed.

## 2. Materials and Methods

The current study utilized a double-blind, randomized, placebo-controlled, parallel-groups design to assess whether 90-day oral supplementation with 200 mg (100 mg twice daily) of Kaneka Ubiquinol™ (Nakanoshima, Kita-ku Osak, Japan) influenced cognition, blood biomarkers related to inflammation and oxidative stress, cardiovascular function, and subjective mood in older adults [49]. The study was completed at the Centre for Mental Health and Brain Sciences (CMHBS) at Swinburne University of Technology (SUT; Melbourne, Australia). The study was approved by the Swinburne University Human Research Ethics Committee (SUHREC; 2018/323) and conducted in compliance with the International Council for Harmonization Good Clinical Practice guidelines. All procedures were completed after participants provided written informed consent. The trial was registered on the Australian New Zealand Clinical Trials Registry (ACTRN12618001841268; https://anzctr.org.au/Trial/Registration/TrialReview.aspx?ACTRN=12618001841268, accessed on 19 June 2026) on 12 November 2018. Reporting of this study follows the CONSORT 2025 statement [50]. The completed checklist and participant flow diagram are provided as Supplementary Materials.

### 2.1. Participants

Participants in the current study were males and females aged 60 years and over residing in metropolitan Melbourne and the surrounding regions. Prospective participants were recruited through advertisements including the SUT website, Australian Clinical Trials website, an internal database of former CMHBS study participants, flyers, as well as via professional services specializing in clinical trial recruitment. Table 1 outlines inclusion/exclusion criteria for the trial.

The target sample size was determined via power analysis conducted with G*Power 3.1.9.2. This analysis indicated that, utilizing repeated-measures analysis of variance (ANOVA) with two treatment groups and two primary time points, a significance threshold *p* < 0.05, and an 80% probability of detecting a small-to-medium effect size (Cohen’s f = 0.14) for time-by-treatment group interaction, a final sample of 104 participants was required. The study aimed to recruit a total of 128 participants, assuming a 20% attrition rate, thereby allowing a final sample of 104 participants to complete the study. After completion of the trial the number of participants with complete data was less than was expected as per the original power analysis. Analysis was then amended to use analysis of covariance (ANCOVA; see Section 2.6. Statistical Analysis) to enhance statistical power. The initial proposed analysis method (repeated-measures ANOVA) was not formerly registered.

### 2.2. General Procedures

Upon expressing interest, potential participants were telephone screened. This screening process included questions regarding inclusion and exclusion criteria. For participants deemed likely eligible after the telephone screening, a screening/training visit was organized to occur at SUT and commenced with participants providing written informed consent. During that visit, qualified trial staff discussed the trial with the participants and answered any remaining questions participants had prior to commencing the study. All inclusion and exclusion criteria were reviewed, including all outstanding criteria which could only be assessed in person. Demographic information including a detailed medical history, height and weight was collected. Participants were then familiarized with all testing procedures.

Baseline testing was conducted within 14 days of the screening/training visit. Participants were instructed to refrain from eating after 10:00 p.m. the night before (only water was allowed), avoid alcohol for 24 h prior, and abstain from vigorous physical activity for at least 12 h before testing. Upon arrival at SUT, participants provided a fasting blood sample and underwent cardiovascular assessments using the SphygmoCor XCEL device (Model XCEL; AtCor Medical, Sydney, Australia). Participants then received a standardized breakfast (cereal and/or wholewheat toast; no tea or coffee was provided) before completing all cognitive and mood assessments, as outlined in Section 2.5 below (Table S1 provides an overview of study assessments across testing visits, with a detailed overview of trial-related outcomes also provided in the Supplementary Methods).

At the conclusion of the baseline visit, participants were randomized to receive either ubiquinol or a matched placebo. Participants were provided with a 90-day supply of their assigned treatment (plus additional doses as a 14-day buffer). Instructions on when and how often to consume the treatment were also provided alongside a diary for participants to record when they consumed their daily treatment.

**Figure 1:**
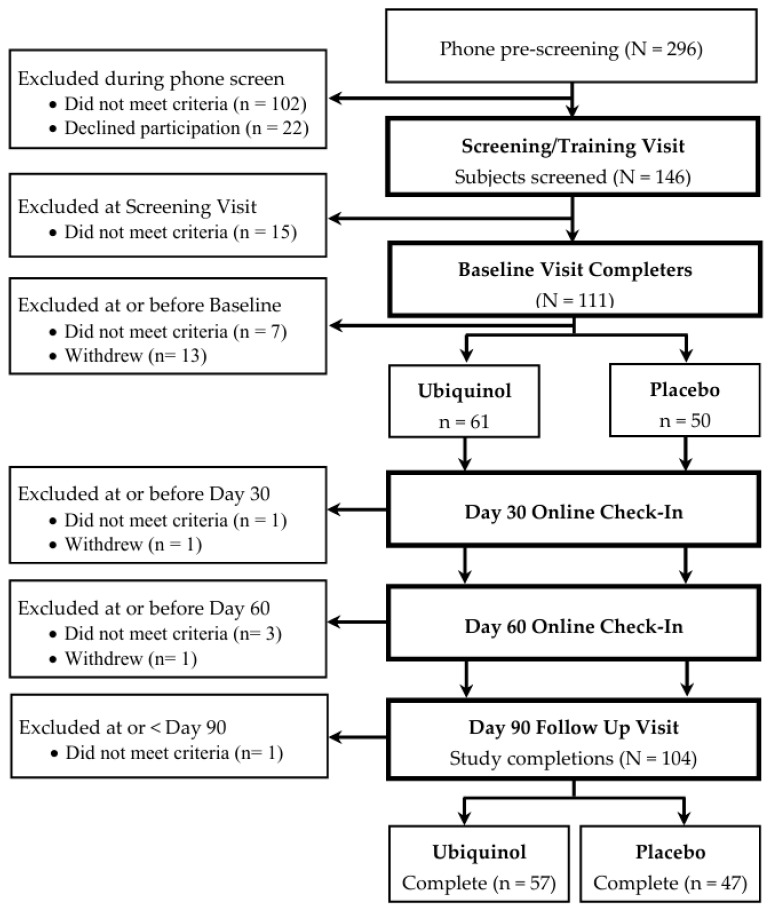
Participant flow during the trial.

The final testing session occurred 90 days after the initiation of supplementation, following the same protocol as the baseline visit. At the conclusion of each testing session (in-person as well as during the online remote check-in sessions scheduled at 30 and 60 days), participants were screened for adverse events (AEs) or serious adverse events (SAEs) through direct questioning and review of participants’ responses during the symptoms checklist (see Supplementary Methods). Any clinically significant symptoms not attributed to pre-existing medical conditions were documented as AEs. If a participant experienced an AE or SAE that persisted beyond the final testing day, consent was obtained for continued follow-up until resolution. Figure 1 provides an outline of participant flow throughout the trial.

### 2.3. Investigational Product

The investigational product, Kaneka Ubiquinol™, was a soft capsule containing 100 mg of ubiquinol emulsified with diglycerol monooleate, rapeseed oil, soy lecithin, and beeswax. A matched appearance placebo capsule was used, containing the same ingredients without the ubiquinol. Participants consumed one capsule at the same time twice daily with food for a total daily dose of 200 mg ubiquinol.

### 2.4. Randomization and Blinding

Randomization of participants to treatment groups in the current study was completed by a CMHBS staff member who was not involved with the current study. Assignment to a treatment group (designated as A or B; ubiquinol and placebo, respectively) occurred via random allocation using a computer random number generator. A CMHBS staff member not involved with the current trial also maintained the key to treatment allocation codes which were not to be accessed except in an emergency such as when management of a SAE required knowledge of the treatment administered.

### 2.5. Outcomes and Measures

The primary outcomes of this study were the memory and processing-time composite scores, with calculation being informed using principal component analysis (PCA). Refer to Section 2.6 for specific information as to how PCA was used in the calculation of these composite scores. Secondary outcomes included assessments of cognition (response accuracy or response time on individual tasks), blood biomarkers specifically related to CoQ10 status, oxidative stress and inflammation, liver enzymes and other fasting blood measures, brachial and aortic (i.e., central) cardiovascular function, as well as questionnaire data regarding subjective memory impairment and mood. As each of these measures were primarily assessed at baseline and after 90 days of treatment, analysis was limited to data collected at these time points (except in the case of the symptoms checklist and testament safety reporting).

#### 2.5.1. Cognition and Mood Assessments

Cognitive function was assessed using a comprehensive battery of validated measures. Self-reported memory concerns were assessed using the Memory Complaint Questionnaire (MAC-Q; range 7–35), with higher scores indicating worse perceived memory [51], and the Prospective and Retrospective Memory Questionnaire (PRMQ; range 16–80), with higher scores indicating more frequent memory failures [52]. Mood was evaluated using the Profile of Mood States (POMS), with higher scores reflecting greater mood disturbance (with the exception of vigor-activity) [53]. Verbal memory was assessed using the Verbal Paired Associates (VPA I and II) and Logical Memory (LM I and II) subtests from the Wechsler Memory Scale—Fourth Edition (WMS-IV) [54], and the Rey Auditory Verbal Learning Test (RAVLT), with higher scores on these tests indicating better memory performance [55]. Attention and working memory were assessed using digit span (DS) from the Wechsler Adult Intelligence Scale—Third Edition (WAIS-III) [56], with higher scores indicating better performance. Processing time was assessed using the Digit Symbol Substitution Test (DSST), with higher scores indicating better performance [57], the Trail Making Test Parts A and B (TMT-A, TMT-B), where shorter completion times reflected better performance [58], and Inspection Time (IT), where shorter stimulus durations were required to achieve 80% accuracy, which was indicative of faster processing times. Further details are provided in Section S1.2 of the Supplemental Methods.

#### 2.5.2. Cardiovascular Measures

Cardiovascular function was assessed using non-invasive measures of blood pressure and arterial stiffness. Brachial and aortic blood pressures were measured using the SphygmoCor XCEL device (AtCor Medical, Sydney, Australia), with higher pressures reflecting elevated cardiovascular load. Peripheral arterial stiffness was assessed via Augmented Pressure (AP) and Augmentation Index (AiX), and aortic stiffness was measured using carotid–femoral pulse wave velocity (cfPWV), with higher cfPWV values indicating greater arterial stiffness [59]. Participants rested in the supine position prior to measurements, and all assessments were conducted according to standardized protocols. Further details are provided in Section S1.3 of the Supplemental Methods.

#### 2.5.3. Biochemical Measures

Participants provided a morning fasting blood sample (fasting from 10:00 p.m. the night before testing which could commence from 8:00 a.m. the following day), collected by a registered nurse or venipuncture technician during the baseline and 90-day testing visits. Samples for assessing plasma CoQ10, High-Sensitivity C-Reactive Protein (HsCRP; higher scores reflect greater inflammation), liver enzymes and other fasting blood measures were analyzed by Australian Clinical Labs (ACL; Melbourne, Australia). Conversely, samples for assessing Diacron-Reactive Oxygen Metabolites (D-ROMS; higher scores reflect greater oxidative stress) and Glutathione Peroxidase (GPx; higher scores reflect greater antioxidant capacity) were analyzed at the Baker Heart and Diabetes Institute (Baker HDI; Melbourne, Australia). Further details are provided in Section S1.4 of the Supplemental Methods.

### 2.6. Statistical Analysis

All data analyses were completed using R (version 4.5.1) and IBM SPSS Statistics (version 28). To reduce the data being right-skewed and alleviate the influence of extreme observations, timed cognitive test variables were log-transformed. Principal component analysis (PCA) was applied to derive two composite scores for reflecting cognitive performance. Originally, composite scores for cognitive performance were to be derived from data collected from a computerized cognitive testing battery. However, due to unforeseen circumstances, these data were lost. As such, PCA was applied to alternate cognitive tests utilized in the trial (outlined in Section 2.5.1) for calculating composite scores.

The first composite score was characterized by high loadings (>0.70) on recall and recognition-based data from the RAVLT (mean immediate recall, delayed recall), LM (immediate recall, delayed recall, delayed recognition), and VPA (immediate recall, delayed recall, delayed recognition) tasks and was designated as the memory composite score. The second composite score, reflecting high loadings (>0.6) from tasks emphasizing processing time, including the IT task, TMT A and B tasks, and the DSST, was designated as the processing-time composite score. To maximize participant data, composite scores were only computed using data from the cognitive tasks which demonstrated a high loading onto these composites during the PCA. Composite scores were standardized as z-scores (mean = 0, standard deviation = 1). A higher memory composite score reflects greater memory performance; conversely, a higher processing-time composite score indicates worse (i.e., slower) performance. The memory and processing-time composite scores were the primary outcomes of this study.

Group differences in cognitive performance, blood biomarkers, cardiovascular function, subjective memory, and mood after 90-day supplementation (i.e., study completion) were assessed using ANCOVA. Each ANCOVA included age, sex, and baseline outcome score as covariates. A complete case analysis approach was employed, whereby participants who completed the trial but nonetheless had missing data at either baseline and/or 90 days were excluded. Follow-up analysis also assessed whether co-varying for statin use (Yes, No) modified results from the original model. Differences between treatment groups were considered significant at the *p* < 0.05 level.

Linear regressions were performed for each treatment group separately to examine how changes in plasma CoQ10, D-ROMS, and GPx, as well as serum HsCRP predicted cognitive performance (i.e., memory and processing-time composite scores) at the final study visit. All models included baseline cognitive performance, as well as participant age, sex, and education. Significance was set at *p* < 0.05. Plasma CoQ10, D-ROMS, and GPx scores were initially divided by 1000 prior to analysis. This only changed the scale of these measures to improve ease of interpretation of results.

## 3. Results

### 3.1. Participant Demographics

A total of 296 prospective participants were screened via telephone. However, only 146 passed and subsequently attended the in-person screening visits at SUT. Of these 146 participants, 131 were deemed eligible to participate following in-person screening, though 20 were either withdrawn or subsequently violated inclusion criteria leaving only 111 participants to attend and complete baseline testing (Figure 1). Initially, 61 participants were randomized to receive Ubiquinol, while the remaining 50 received the placebo, though only 104 participants (95% of initial population; ubiquinol, n = 57; placebo, n = 47) completed the trial. See Table 2 for an overview of demographics of participants who were randomized to treatment in the trial.

As indicated in Table 2, there were no significant differences in baseline demographics between the groups when considering all available data (this was also apparent when analysis was restricted to participants who completed the trial). The only measurement significantly different between groups at baseline was digit span, with the ubiquinol group outperforming the placebo group. See Table 3 for baseline cognition and mood outcomes (trial completers only) and Table 4 for baseline cardiovascular function and blood biomarkers (trial completers only; see also Supplementary Table S2).

### 3.2. Primary Outcomes

There was no difference between the ubiquinol and placebo groups for memory or processing-time composite scores at study completion (after 90 days of supplementation) when covarying for baseline performance, age and sex (Table 5). Additionally covarying for the use of statins did not change results.

### 3.3. Secondary Outcomes

#### 3.3.1. Cognitive Function

Treatment effects were also examined for each individual cognitive task. There were no between-group differences in performance at study completion (after 90 days of supplementation) indicating any beneficial effect of ubiquinol (Table 5). The only instance where a significant difference in performance at 90 days (controlling for baseline performance, sex, and age) was observed was on the digit span task, but in a direction favoring the placebo. Additionally covarying for the use of statins did not change results.

There were no group differences in prospective and retrospective memory at study completion (Table 6). Similarly, no group differences in other subjective memory subscales derived from the PRMQ were identified at study completion. Additionally covarying for the use of statins did not change results.

#### 3.3.2. Subjective Mood

As shown in Table 7, there were no group differences in subjective mood, assessed using the POMS at study completion. Additionally, the use of statins did not change results.

#### 3.3.3. Cardiovascular Function

There were no group differences in brachial or aortic blood pressures assessed at study completion (Table 8), nor were there any group differences in measures of peripheral (AP, AiX) or aortic (cfPWV) arterial stiffness. Additionally covarying for the use of statins did not change results.

#### 3.3.4. Blood Biomarkers

**Figure 2:**
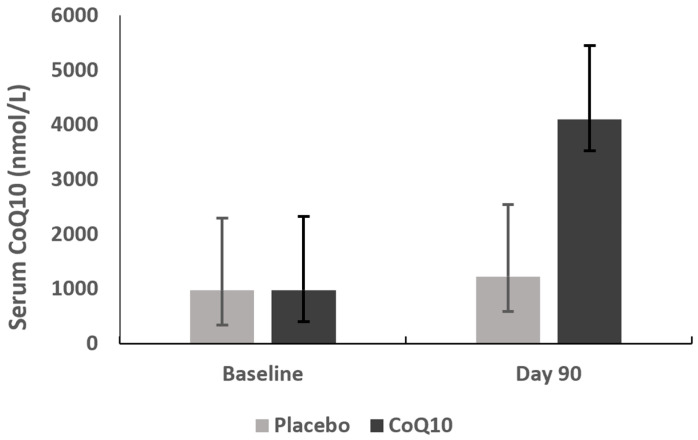
Mean plasma CoQ10 (mean ± SD) levels before and after treatment.

The only blood biomarker significantly different between the ubiquinol and placebo groups at 90 days was plasma CoQ10 (Table 8; See also Supplementary Table S3). The CoQ10 treated group had a significantly greater plasma CoQ10 concentration at the end of the study compared to the placebo group (*p* < 0.001). Numerically, this represents a fourfold greater tissue availability of plasma CoQ10 at study end. Figure 2 presents the mean plasma CoQ10 at baseline and 90 days for both groups (limited to those included in the final intervention analysis). Additionally covarying for the use of statins did not change results.

#### 3.3.5. Regression Analyses

**Figure 3:**
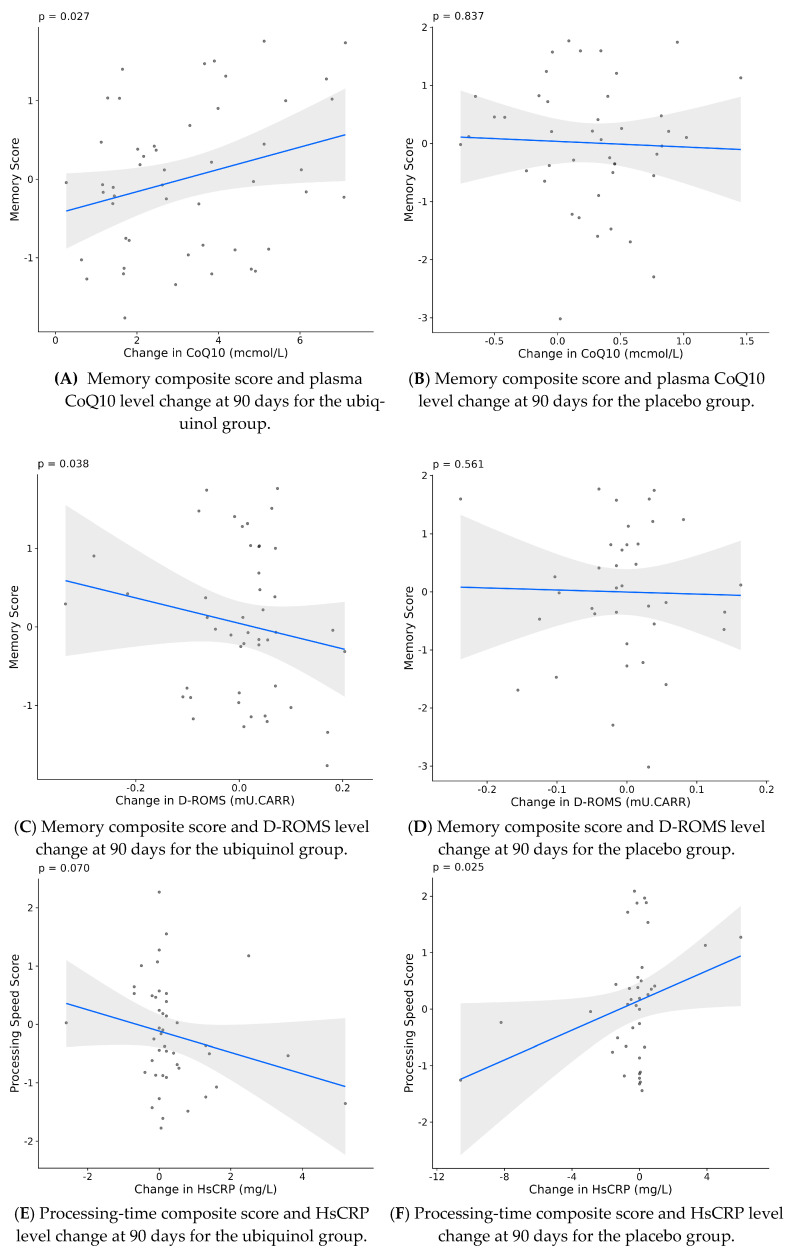
Change in CoQ10 levels, D-ROMS, and HsCRP after 90 days of supplementation predicting memory and processing-time composite scores at trial end.

Separate regression analyses were conducted to assess associations between the change in relevant biomarkers (i.e., plasma CoQ10, D-ROMS, serum HsCRP, and GPx) and final-visit memory and processing-time composite scores for each treatment group (Figure 3—shaded areas represent 95% confidence intervals; see also Tables S5 and S6).

For plasma CoQ10, in the ubiquinol group, an increase over time predicted a greater memory composite score at final follow-up (β = 0.08, 95% CI = 0.01, 0.16, *p* = 0.03; Figure 3A) but not in the placebo group (Figure 3B).

For DROMS (oxidative stress), in the ubiquinol group, an inverse relation was observed whereby a lower D-ROMS over time predicted greater memory composite score at final follow-up (β = −1.54, 05% CI = −2.98, −0.09, *p* = 0.04; Figure 3C), but not in the placebo group (Figure 3D).

For HsCRP (inflammation), in the placebo group, a greater increase over time predicted increased processing time (worse cognition) at final follow-up (β = 0.07, 95% CI = 0.01, 0.13, *p* = 0.03; Figure 3F). However, in the ubiquinol group this negative association was nullified (Figure 3E).

For GPx a change in plasma over time was found not to predict memory or processing-time composite score at trial end in either group (see also Supplementary Tables S5 and S6 and Supplementary Figure S2).

### 3.4. Treatment Safety

Table 9 presents the adverse events (AEs) reported during the study and their classification in relation to treatment. Overall, 144 AEs were documented, comprising 71 events in the ubiquinol group and 73 in the placebo group. Within the ubiquinol group, 37 AEs were assessed as unrelated to treatment, 28 as unlikely to be related, and six as possibly related. No events were considered probably or highly probably related to treatment. In the placebo group, 36 AEs were classified as unrelated, 25 as unlikely to be related, and 12 as possibly related. Similarly, no events in that group were judged to be probably or highly probably related to treatment. No serious adverse events (SAEs) were reported that were deemed to be related (i.e., ‘possible’, ‘probable’, or ‘highly probable’) to the treatments administered in the trial. Three adverse reactions (ARs) were reported in the placebo group.

When assessed using the Symptoms Checklist at baseline and Day 90, there were no significant differences in symptom changes between participants who received ubiquinol and those who received placebo (see Supplementary Table S4 and Figure S1).

## 4. Discussion

The aim of the current study was to determine if 90 days of supplementation with 200 mg of ubiquinol (100 mg twice daily) could influence cognitive function in older adults. While 90 days of ubiquinol supplementation facilitated a marked increase in plasma CoQ10 levels, indicating high bioavailability, it did not facilitate greater cognitive performance relative to placebo at the end of the trial. Furthermore, there were no significant between-group differences in secondary outcomes including oxidative stress, inflammation, blood pressure (or other measures of cardiovascular health), subjective memory or mood at the end of the trial.

However, regression analyses indicated a positive association between increased plasma CoQ10 level over the intervention period and higher cognitive performance (memory composite score) at the end of the trial, specifically in those treated with ubiquinol. Likewise, greater reductions in oxidative stress (D-ROMS) over the intervention period predicted higher memory performance (memory composite score) at the end of the trial, specifically in those in the intervention group. Interestingly, while greater inflammation (HsCRP) levels over time predicted slower cognitive processing (processing-time composite score) in the placebo group, that negative association was nullified in the ubiquinol group.

The literature pertaining to cognitive benefits of CoQ10 supplementation in humans is mixed, with benefits reported primarily in disease cohorts [26]. Importantly, only two studies have investigated possible cognitive effects following CoQ10 supplementation in cognition in healthy adults. In the first, Kennedy et al. [30] administered 4.5 mg of CoQ10 per day (8 weeks) as part of a multi-nutrient formulation, but no effects upon cognition relative to placebo were observed. In the second trial, Kinoshita et al. [31] discovered that when 100 mg of ubiquinol per day was administered for 34 weeks, there were no beneficial cognitive effects compared to placebo reported except in a small subgroup. An important distinction between our study and Kinoshita et al.’s [31] is that despite a higher administered dose (i.e., 200 mg daily) the current study utilized a much shorter intervention period (approximately 13 vs. 34 weeks). It is plausible that the current intervention period was insufficient to induce any major cognitive differences despite a higher administered dose.

Interestingly, in the present study, regression analysis determined that elevated plasma CoQ10 levels over time were predictive of better cognitive performance (memory composite score) at the end of the study but only in the group that received ubiquinol. Furthermore, in the ubiquinol group but not in the placebo group, a negative association between change in oxidative stress (D-ROMS) and memory performance at the final visit indicates that lower oxidative stress induced by ubiquinol may predict better cognitive function. Thus, elevating CoQ10 bioavailability through ubiquinol supplementation, and potentially mitigating oxidative stress, may contribute to better cognitive performance in older adults, though additional clinical trials utilizing larger participant samples and longer intervention periods are required to confirm this.

While we did not observe any significant group differences in cognitive function at study end, several elements of the current study ultimately limit conclusions regarding the efficacy of ubiquinol for modifying trajectories of cognitive decline over time in older adults. For example, in the current study, participants were screened using the MAC-Q and required a score of 19 or more. Such a score would have allowed participation of individuals who believed that their memory had not declined since young adulthood or at least very mildly overall, as well as those with a strong sense of subjective memory decline. The use of a higher cut-off score would have facilitated the recruitment of participants with a greater sense of subjective impairment (potentially reflecting elevated risk of cognitive impairment) which in turn may have increased the likelihood of cognitive benefits being experienced in response to ubiquinol. Moreover, participants were not screened for mild cognitive impairment (i.e., performing poorer than expected for their age and education level with intact activities of daily living). Greater decrements in cognitive performance over time by MeDi adherence and by OM-3 PUFA supplementation have been reported in adults with documented MCI relative to cognitively normal adults [60,61], indicating that an MCI group may be a more appropriate sample for intervention studies such as ours. Indeed, the enhanced risk of decline over time was part of the reason patients with MCI were specifically recruited for the VITACOG study [62], which subsequently provided strong evidence that two years of daily supplementation with vitamin B6, B12, and folate mitigated decrements in brain structure and cognitive function [62,63,64].

An earlier study by García-Carpintero et al. [37] investigated possible cognitive effects following 12 months of ubiquinol supplementation (200 mg daily) in older adults with MCI but failed to identify any significant benefits upon cognitive performance. Considering earlier works having reported cognitive decline in MCI adults over periods of 12 [61] to 18 months [60], the absence of cognitive benefits detected by Garcia-Carpintero et al. [37] may be due to insufficient statistical power (total sample size = 69) for detecting treatment-related cognitive effects, rather than limitations related to intervention duration. Considering the current study utilized an even shorter intervention period (approximately 13 weeks), the current trial was likely more appropriate for detecting short-term cognitive improvements (which were absent in this study) and ill equipped for detecting treatment effects upon longer-term cognitive trajectories.

Future studies with ubiquinol (or other forms of CoQ10) should incorporate larger samples of older adults with conditions such as MCI, which is associated with a greater risk of subsequent cognitive decline over time. Longer intervention periods are also advised (e.g., 18 months or more) to allow for sufficient time for age-related cognitive change and prodromal neurodegenerative disease to become apparent and subsequent changes to these trajectories in response to ubiquinol supplementation to be detectable.

### 4.1. Targeting Mechanistic Pathways for Modifying Cognitive Function

The absence of differential treatment effects upon cognitive function in the current study may also be due to the current intervention (200 mg of ubiquinol for 90 days) not inducing any beneficial changes in biological factors known to predict poorer cognitive performance (e.g., elevated oxidative stress, inflammation, and elevated blood pressure). As discussed previously, several meta-analyses have reported that CoQ10 supplementation can improve markers associated with oxidative damage, while increasing markers indicative of greater antioxidant capacity [40,42,43], as well as reducing inflammatory biomarkers [43,44,45]. However, subgroup analyses in many of these meta-analyses indicate that the benefits of CoQ10 supplementation on these factors is dependent on participant health status. For example, significant improvements in total antioxidant capacity (TAC) following CoQ10 intervention were evident in patients with diabetes but not in non-diabetics [40] or healthy adults [41]. While our study utilized a different measure of oxidative stress (i.e., D-ROMS) than these earlier meta-analyses, the absence of therapeutic benefits to these measures may still be due to the current study restricting participation to healthy older adults (no participants had a diagnosis of cardiovascular disease or diabetes, and our sample had a low prevalence of hypertension (the ubiquinol group included 12 participants (21%) and the placebo group included 12 participants (25%) at study entry), and infrequent use of cholesterol lowering medications (the ubiquinol group included 10 participants (17%) and the placebo group included nine participants (19%) at study entry). As indicated in Table 4 and Table 8, plasma D-ROMS levels indicated a low level of oxidative stress in the current cohort. Selection of a sample with enhanced oxidative stress at study entry may be more likely to demonstrate a benefit from ubiquinol and subsequently demonstrate cognitive effects in response to ubiquinol’s antioxidant effect. Similarly, in one meta-analysis [45], CoQ10 therapy was found to be efficacious for lowering HsCRP levels in healthy adults. However, it should be noted that these effects were reported in only two studies [65,66], utilizing different doses (100 mg or 300 mg per day, respectively), intervention periods (8 weeks or 2 weeks, respectively), and participants (both involved only males with a mean age less than 20 years) compared to the current study. As such, differences between these earlier studies’ methodologies and the current study limit the comparability of results.

Congruent with the above meta-analyses for oxidative stress and inflammation, the therapeutic benefits of CoQ10 upon blood pressure may be more apparent in ‘unhealthy’ adults. Earlier meta-analyses [47,67,68] were limited to adults with conditions such as hypertension or cardiometabolic disorders (e.g., diabetes or dyslipidemia), though a recent meta-analysis (incorporating data from 45 clinical trials) included subgroup analysis contrasting adults with and without preexisting health conditions [46]. In this latter meta-analysis, while CoQ10 intervention significantly reduced blood pressure, these effects were only apparent in adults with pre-existing health conditions and not those deemed healthy (i.e., no pre-existing health conditions).

Considering data from the above meta-analyses, CoQ10 supplementation appears to primarily benefit oxidative stress, inflammation, and hypertension in ‘unhealthy’ samples rather than in those deemed healthy. Subsequently, cognitive benefits in response to ubiquinol (or other CoQ10 variants) may be more likely to occur when administered to older adults diagnosed with conditions predisposing them to elevated oxidative stress, inflammation, and/or higher blood pressures—factors predictive of cognitive decline.

### 4.2. Strengths and Limitations

The current study has several strengths. Firstly, the sample size recruited for the current study is larger than that of most human trials assessing cognitive effects in response to CoQ10 (including ubiquinol) supplementation [26]. This is also true of studies exploring whether CoQ10 benefits oxidative stress [41], inflammation [45] or cardiovascular function [46]. Secondly, the current study examined whether ubiquinol reduced oxidative stress and inflammation, and cardiovascular function in older adults, whereas other studies assessing such effects typically involve younger-aged samples [41,45,46]. This might contribute to a better understanding of how ubiquinol (or other CoQ10 forms) could support cognitive function via mitigating risk factors known to predict poorer cognitive function in later life. Finally, we also assessed aortic blood pressures, whereas other works assessing cardiovascular effects of nutritional interventions typically assess only peripheral or brachial blood pressures. Earlier works have indicated that aortic blood pressure may be more amenable to nutritional interventions than peripheral pressures [69,70,71].

However, the current study also has several limitations which should be considered when interpreting the results of this study. As discussed above, participants typically did not possess health conditions (e.g., diabetes, dyslipidemia, cardiovascular dysfunction) predisposing them to elevated oxidative stress, inflammation, or elevated blood pressure—factors that are readily modifiable via CoQ10 supplementation which in turn could underpin potential cognitive benefits. Another potential limitation is that the sample was heterogenous regarding the level of current cognitive impairment and/or risk of future decline. This may have reduced the sensitivity of the current study for detecting cognitive effects following ubiquinol supplementation. Finally, while the current study utilized a sample typically larger than other studies assessing the effect of CoQ10 (or its analogs) upon cognitive function [26], it is possible that the current study was still underpowered to detect small-to-moderate treatment effects over a relatively short observation period (90 days). Future studies should involve doses of CoQ10 that are clinically effective for modifying cognitive function or mechanisms (e.g., oxidative stress, inflammation, cardiovascular function) underpinning cognitive degradation. Likewise, future studies should consider incorporating larger samples of older adults, potentially those with MCI, as well as longer intervention periods to explore whether CoQ10 supplementation enhances cognitive function or modifies the trajectory of cognitive decline. Such studies could also target factors such as elevated oxidative stress, inflammation, or blood pressure to determine the extent to which modification of these factors underpins improved cognitive trajectories in older adults.

## 5. Conclusions

In the current study 200 mg of ubiquinol (100 mg twice daily) was administered for a period of 90 days to older adults with essentially normal cognition. While the intervention elevated plasma CoQ10 levels, no group differences favoring the ubiquinol group were observed for composite measures of memory or processing time.

However, post hoc analyses did indicate that in the ubiquinol group, but not in the placebo group, increased plasma CoQ10 was predictive of greater memory performance at study end. Furthermore, in the ubiquinol group, but not in the placebo group, there was an association between reduced oxidative stress over time and improved cognitive performance. We believe that these findings are indications of a potential benefit on cognition of raising coenzyme levels by ubiquinol supplementation that merits further investigation in a new trial. This would be conducted over a longer period, in an adequate number of subjects with mild cognitive decline or increased propensity for cognitive decline due to elevated risk factors such as oxidative stress.

## Figures and Tables

**Table 1:** Study inclusion and exclusion criteria.

Inclusion Criteria	Exclusion Criteria
Non-smoking males and females aged 60 years and over.	Psychiatric disorder requiring treatment in the previous two years. Treatment includes prescription of antidepressant, antipsychotic or other long-term medication and/or referral for long-term psychotherapy. It does not include brief interventions for normal life events such as exam anxiety or bereavement.
2. Willing and able to provide written informed consent.	2. Neurological conditions including epilepsy, Parkinson’s disease, Huntington’s chorea and myasthenia gravis.
3. Understands and is willing and able to comply with all study procedures.	3. History of dementia, stroke and other neurological conditions.
4. Fluent in written and spoken English.	4. Head trauma with loss of consciousness in the previous 6 months.
5. Must be in general good health, defined by the absence of exclusion criteria.	5. History of Type I diabetes (insulin dependent) or Type II diabetes on treatment (Type II diabetes and prediabetes treated with diet alone is not an exclusion).
6. Normal or corrected to normal vision.	6. Cardiovascular disease.
7. No recent history (past five years) of chronic severe illness (longer than six weeks).	7. Current endocrine, gastrointestinal or bleeding disorders.
8. Willing to maintain habitual diet (including caffeine and alcohol) and physical activity patterns throughout the study period.	8. Uncontrolled hypertension (systolic blood pressure > 160 mmHg or diastolic pressure > 90 mmHg).
9. Willing and able to comfortably abstain from caffeine for 10 h prior to and throughout the test visits.	9. If female, pregnant or lactating.
10. Willing to abstain from alcohol for 24 h and vigorous physical activity for 12 h prior to all study visits.	10. Alcohol or drug abuse (past or present).
11. Willing and able to provide two blood samples.	11. Other disorders affecting food metabolism, such as food allergies or intolerances.
12. Score of 19 or above on the Memory Complaint Questionnaire (MAC-Q).	12. Taking any (and not willing to abstain for the duration of the trial): a. Coenzyme Q10 b. Dietary and herbal supplements known to influence cognition (includes a 4-week washout preceding baseline). c. Antithrombotic medications d. Cholinergic system medications e. Anxiolytics f. Antidepressants g. Sedatives h. Illicit drugs
13. Not currently participating in any other clinical trial involving an investigational product in the last 4 weeks.	13. Current regular alcohol use exceeding 14 standard drinks per week for women, and 28 standard drinks per week for men.
14. Has access to an internet device capable of online testing.	14. Current moderate or severe alcohol misuse disorder as defined in DSM5.
	15. Current substance misuse disorder as defined in DSM5 (including misuse or prescription drugs).
	16. Participant under administrative or legal supervision.
	17. Allergy to the investigational product or formulation.

**Table 2:** Demographics of randomized participants.

	Ubiquinol (n = 61)	Placebo (n = 50)	*p* Value
Age, mean (SD)	67.6 (5.0)	68.7 (5.9)	0.29
Gender (male), n (%)	30 (49.2)	21 (42.0)	0.47
Ethnicity, n (%)			0.39
Caucasian	58 (95.1)	48 (96.0)	
Asian	3 (4.9)	1 (2.0)	
Other	0 (0.0)	1 (2.0)	
Highest Education Level, n (%)			0.71
Primary	0 (0.0)	1 (2.0)	
Secondary	14 (23.0)	12 (24.0)	
Tertiary	30 (49.2)	25 (50.0)	
Postgraduate	17 (27.9)	12 (24.0)	
Years of Education, mean (SD)	16.1 (4.0)	15.5 (3.7)	0.35
Use of Statins, n (%)	10 (16.4)	10 (20.0)	0.59
Use of Anti-Hypertensives, n (%)	13 (21.3)	13 (26.0)	0.48
MAC-Q Score, mean (SD)	24.7 (3.1)	24.9 (2.8)	0.71

Note: One-way ANOVA used to assess difference for continuous variables, Pearson’s X^2^ used for categorical variables. Abbreviations: MAC-Q, Memory Complaints Questionnaire.

**Table 3:** Baseline statistics for measures of cognitive function and mood (study completers).

Parameter	Ubiquinol			Placebo			t	*p*
	n	Mean	SD	n	Mean	SD		
Cognition								
** Memory Composite Score**	54	0.11	0.91	44	0.04	1.06	0.36	0.72
** Processing-Time Composite Score**	54	−0.15	0.94	42	0.15	1.04	−1.46	0.15
LM—I Immediate Recall	54	35.20	7.30	44	34.73	6.06	0.35	0.73
LM—II Delayed Recall	57	21.05	5.83	47	20.09	6.11	0.82	0.41
LM—II Delayed Recognition	57	19.07	2.43	47	19.23	2.15	−0.36	0.72
VPA—Immediate Recall	57	33.12	8.51	47	31.55	10.11	0.86	0.39
VPA—Delayed Recall	57	10.21	2.40	47	9.91	2.98	0.56	0.58
VPA—Delayed Recognition	57	38.96	1.30	47	38.28	2.52	1.70	0.09
RAVLT—Immediate Recall (mean)	57	10.13	1.69	47	9.97	2.08	0.44	0.66
RAVLT—Delayed Recall	57	9.33	3.65	47	8.11	4.33	1.54	0.13
DSST	57	53.14	9.17	47	53.79	8.83	−0.36	0.72
DS	57	19.60	4.00	47	17.94	3.94	2.12	0.04 *
TMT-A (ln transformed)	56	10.08	0.27	47	10.15	0.31	−1.30	0.20
TMT-B (ln transformed)	55	10.80	0.36	43	10.94	0.39	−1.80	0.08
IT (ln transformed)	56	4.59	0.31	46	4.66	0.39	−0.93	0.35
PRMQ Total	56	59.64	8.04	47	59.85	7.29	−0.14	0.89
PRMQ Short-Term Memory	56	28.89	4.56	47	29.32	3.81	−0.51	0.61
PRMQ Long-Term Memory	57	30.72	4.08	47	30.53	3.90	0.24	0.81
PRMQ Self-Cued	56	28.46	4.31	47	28.47	4.15	−0.01	1.00
PRMQ Environment-Cued	56	31.18	4.09	47	31.38	3.75	−0.26	0.79
PRMQ Prospective	56	29.18	4.24	47	29.21	4.27	−0.04	0.97
PRMQ Retrospective	56	30.46	4.37	47	30.64	3.70	−0.22	0.83
Mood (POMS)								
Tension Subscale	57	3.42	2.56	47	3.43	3.15	−0.01	0.99
Depression Subscale	57	2.96	4.30	47	3.47	4.65	−0.57	0.57
Anger Subscale	57	2.89	3.38	47	3.28	4.10	−0.52	0.60
Fatigue Subscale	57	3.75	3.38	47	4.57	4.29	−1.09	0.28
Confusion Subscale	57	4.04	2.78	47	4.51	2.83	−0.86	0.39
Vigor Subscale	57	20.39	5.13	47	19.00	5.99	1.27	0.21
Total Mood Disturbance	57	−3.32	13.88	47	0.26	19.31	−1.10	0.28

Note: Levene’s Test of Equality of Variances was significant for Verbal Pairs—Delay Recognition, RAVLT—Immediate Recall (mean) and RAVLT—Delayed Recall; subsequently, *t*-tests were performed with equal variances not being assumed. Abbreviations: LM, Logical Memory; VPA, Verbal Paired Associates; RAVLT, Rey Auditory Verbal Learning Test; DSST, Digit Symbol Substitution; DS, Digit Span; TMT, Trail Making Test; IT, Inspection Time; PRMQ, Prospective and Retrospective Memory Questionnaire. * *p* < 0.05.

**Table 4:** Baseline statistics for measures of cardiovascular function and blood biomarkers at (study completers).

Parameter	Ubiquinol			Placebo			t	*p*
	n	Mean	SD	n	Mean	SD		
Cardiovascular Function								
Brachial Systolic Pressure (mmHg)	57	126.68	13.16	47	126.70	12.58	−0.01	0.99
Brachial Diastolic Pressure (mmHg)	57	74.93	7.94	47	74.04	9.31	0.52	0.60
Aortic Systolic Pressure (mmHg)	57	116.56	11.38	47	116.64	10.82	−0.04	0.97
Aortic Diastolic Pressure (mmHg)	57	75.89	8.04	47	74.98	9.32	0.54	0.59
Aortic Pulse Pressure (mmHg)	57	40.67	8.55	47	41.66	8.72	−0.58	0.56
Mean Arterial Pressure (mmHg)	57	90.49	8.97	47	89.60	9.32	0.50	0.62
Augmentation Pressure (mmHg)	57	11.74	5.39	47	11.40	4.62	0.33	0.74
Augmentation Index (%)	57	28.18	10.14	47	27.04	8.92	0.60	0.55
cfPWV (m/s)	53	10.93	1.68	43	10.93	1.72	0.00	1.00
Heart Rate (bpm)	57	60.61	9.94	47	58.91	8.18	0.94	0.35
Blood Biomarkers								
Plasma CoQ10 (nmol/L)	56	977.16	338.44	47	962.89	389.56	0.20	0.84
HsCRP (mg/L)	54	0.96	1.57	47	3.40	13.18	−1.26	0.21
GPx (mmol/min/mL)	54	919.45	353.68	44	929.26	381.41	−0.13	0.90
D-ROMS (U.CARR)	54	269.43	75.29	42	299.14	73.21	−1.94	0.06

Note: Levene’s Test of Equality of Variances was significant for High-Sensitivity C-Reactive Protein; subsequently, *t*-tests were performed with equal variances not being assumed. Abbreviations: mmHg, millimeters of mercury; cfPWV, carotid–femoral pulse wave velocity; CoQ10, Co-enzyme Q10; HsCRP, High-Sensitivity C-Reactive Protein; GPx, Glutathione Peroxidase; D-ROMS, Diacron-Reactive Oxygen Metabolites; U.CARR, Carratelli Units.

**Table 5:** Estimated Marginal Means and effect of treatment on cognitive performance (study completers).

Outcome	90-Day Follow-Up						Effect of Treatment				
	Ubiquinol			Placebo			F	*p*	Mean Difference at 90 Days	95% CI	η^2^
	n	EMM	SE	n	EMM	SE					
**Memory Composite Score**	54	0.03	0.19	44	0.12	0.20	0.68	0.41	−0.09	−0.32, 0.13	0.01
**Processing-Time Composite Score**	48	0.18	0.17	41	0.21	0.18	0.08	0.78	−0.03	−0.24, 0.18	0.00
LM—I Immediate Recall	54	34.75	1.45	44	35.09	1.55	0.15	0.70	−0.34	−2.06, 1.38	0.00
LM—II Delayed Recall	57	23.45	1.70	47	23.76	1.80	0.10	0.75	−0.31	−2.27, 1.65	0.00
LM—II Delayed Recognition	57	19.38	0.63	47	19.48	0.67	0.08	0.77	−0.11	−0.82, 0.61	0.00
VPA—Immediate Recall	57	36.50	2.13	47	36.49	2.26	0.00	0.99	0.01	−2.45, 2.47	0.00
VPA—Delayed Recall	57	10.79	0.60	47	10.92	0.64	0.14	0.71	−0.13	−0.82, 0.56	0.00
VPA—Delayed Recognition	57	38.60	0.53	47	39.08	0.56	2.41	0.12	−0.48	−1.09, 0.13	0.02
RAVLT—Immediate Recall (mean)	57	10.38	0.48	47	10.26	0.51	0.18	0.67	0.12	−0.44, 0.68	0.00
RAVLT—Delayed Recall	57	10.20	1.11	47	10.21	1.18	0.00	0.98	−0.01	−1.31, 1.28	0.00
DSST	57	53.13	2.07	47	53.52	2.19	0.11	0.74	−0.39	−2.76, 1.97	0.00
DS	57	19.12	0.84	47	20.53	0.90	8.12	0.005 **	−1.41	−2.39, −0.43	0.08
TMT-A (ln transformed)	56	10.22	0.07	47	10.25	0.07	0.68	0.41	−0.03	−0.11, 0.05	0.01
TMT-B (ln transformed)	52	10.84	0.09	41	10.81	0.10	0.29	0.59	0.03	−0.08, 0.14	0.00
IT (ln transformed)	52	4.62	0.08	45	4.67	0.09	0.95	0.33	−0.05	−0.15, 0.05	0.01

Note: TMT-A and TMT-B tasks and Inspection Time were originally recorded in milliseconds (ms). Abbreviations: EMM, Estimated Marginal Means; SE, Standard Error; CI, confidence interval; LM, Logical Memory; VPA, Verbal Paired Associates; RAVLT, Rey Auditory Verbal Learning Test; DSST, Digit Symbol Substitution; DS, Digit Span; TMT, Trail Making Test; IT, Inspection Time. ** *p* < 0.01.

**Table 6:** Estimated Marginal Means and effect of treatment on Prospective and Retrospective Questionnaire responses (study completers).

Outcome	90-Day Follow-Up						Effect of Treatment				
	Ubiquinol			Placebo			F	*p*	Mean Difference at 90 Days	95% CI	η^2^
	n	EMM	SE	n	EMM	SE					
PRMQ Total	56	60.56	1.75	47	61.71	1.86	1.26	0.26	−1.15	−3.17, 0.88	0.01
PRMQ Short-Term Memory	56	29.63	1.01	47	29.99	1.07	0.37	0.54	−0.36	−1.53, 0.81	0.00
PRMQ Long-Term Memory	57	30.91	0.87	47	31.80	0.93	3.14	0.08	−0.90	−1.90, 0.11	0.03
PRMQ Self-Cued	56	29.15	0.99	47	30.04	1.05	2.42	0.12	−0.90	−2.04, 0.25	0.02
PRMQ Environment-Cued	56	31.42	0.96	47	31.72	1.02	0.29	0.59	−0.30	−1.41, 0.81	0.00
PRMQ Prospective	56	30.04	1.02	47	30.80	1.08	1.60	0.21	−0.75	−1.94, 0.43	0.02
PRMQ Retrospective	56	30.51	0.95	47	30.94	1.00	0.61	0.44	−0.43	−1.52, 0.67	0.01

Abbreviations: EMM, Estimated Marginal Means; SE, Standard Error; CI, confidence interval; PRMQ, Prospective and Retrospective Memory Questionnaire.

**Table 7:** Estimated Marginal Means and effect of treatment on Profile of Mood States scores.

Outcome	90-Day Follow-Up						Effect of Treatment				
	Ubiquinol			Placebo			F	*p*	Mean Difference at 90 Days	95% CI	η^2^
	n	EMM	SE	n	EMM	SE					
Tension Subscale	57	3.01	0.85	47	3.27	0.90	0.27	0.60	−0.26	−1.23, 0.72	0.00
Depression Subscale	57	4.83	1.09	47	4.87	1.17	0.00	0.95	−0.04	−1.22, 1.14	0.00
Anger Subscale	57	3.11	0.99	47	3.31	1.05	0.12	0.73	−0.20	−1.34, 0.94	0.00
Fatigue Subscale	57	3.22	1.17	47	4.14	1.24	1.83	0.18	−0.92	−2.26, 0.43	0.02
Confusion Subscale	57	4.15	0.64	47	3.63	0.68	1.99	0.16	0.52	−0.21, 1.26	0.02
Vigor Subscale	57	18.69	1.38	47	19.20	1.47	0.40	0.53	−0.51	−2.12, 1.10	0.00
Total Mood Disturbance	57	−1.69	3.55	47	−1.53	3.79	0.01	0.94	−0.16	−4.25, 3.93	0.00

Abbreviations: EMM, Estimated Marginal Means; SE, Standard Error; CI, confidence interval.

**Table 8:** Estimated Marginal Means and effect of treatment on cardiovascular function and blood biomarkers.

Outcome	90-Day Follow-Up						Effect of Treatment				
	Ubiquinol			Placebo			F	*p*	Mean Difference at 90 Days	95% CI	η^2^
	n	EMM	SE	n	EMM	SE					
Brachial Systolic Pressure	53	129.63	3.05	45	128.45	3.24	0.44	0.51	1.19	−2.38, 4.76	0.01
Brachial Diastolic Pressure	53	74.34	1.88	45	74.37	2.00	0.00	0.98	−0.03	−2.25, 2.19	0.00
Aortic Systolic Pressure	52	118.56	2.71	45	117.77	2.88	0.24	0.63	0.79	−2.41, 3.98	0.00
Aortic Diastolic Pressure	52	75.49	1.92	45	75.45	2.04	0.00	0.97	0.04	−2.23, 2.32	0.00
Aortic Pulse Pressure	52	43.20	1.88	45	42.48	2.00	0.42	0.52	0.72	−1.48, 2.93	0.01
Mean Arterial Pressure	52	90.85	2.10	45	90.66	2.24	0.02	0.88	0.18	−2.31, 2.68	0.00
Augmentation Pressure	52	11.80	1.19	45	12.09	1.27	0.16	0.69	−0.29	−1.72, 1.14	0.00
Augmentation Index (%)	52	26.67	2.44	45	27.62	2.59	0.42	0.52	−0.95	−3.88, 1.98	0.01
cfPWV (m/s)	47	11.80	0.44	32	11.94	0.48	0.23	0.63	−0.14	−0.71, 0.44	0.00
Heart Rate (during PWA; bpm)	52	61.07	2.02	45	62.33	2.14	1.09	0.30	−1.26	−3.66, 1.14	0.01
Plasma CoQ10 (nmol/L)	53	3867.90	492.60	45	960.78	525.17	98.96	<0.001 ***	2907.12	2326.70, 3487.54	0.52
HsCRP (mg/L)	51	1.20	1.38	46	1.57	1.47	0.21	0.65	−0.38	−2.02, 1.27	0.00
GPx (mmol/min/mL)	49	769.45	92.14	41	774.10	98.55	0.01	0.94	−4.65	−117.81, 108.51	0.00
D-ROMS (U.CARR)	49	310.97	28.69	39	314.91	30.97	0.05	0.83	−3.94	−40.17, 32.30	0.00

Notes: All pressure variables measure in millimeters of mercury (mmHg). Abbreviations: EMM, Estimated Marginal Means; SE, Standard Error; CI, confidence interval; cfPWV, carotid–femoral pulse wave velocity; PWA, pulse wave analysis; bpm, beats per minute; CoQ10, Co-enzyme Q10; HsCRP, High-Sensitivity C-Reactive Protein; GPx, Glutathione Peroxidase; D-ROMS, Diacron-Reactive Oxygen Metabolites; U.CARR, Carratelli Units. *** *p* < 0.001.

**Table 9:** Adverse events and adverse reactions for duration of the clinical trial.

Adverse Events	Ubiquinol	Placebo
	Events Related to Treatment	Events Related to Treatment
Number of Adverse Events	6	12
Headache	1	2
Itchiness	1	1
Gastrointestinal Condition	1	2
Sleep Issues	1	2
Dizziness		3
Dry Mouth	2	
Weight Gain		1
Blood Biomarkers Outside of Normal Range		1
Number of Adverse Reactions	0	3
Itchiness		1
Gastrointestinal Condition		1
Sleep Issues		1

Note: Events related to treatment only include AEs and ARs deemed to be related to the treatment administered (i.e., ‘possible’, ‘probable’, or ‘highly probable’). Abbreviations: AE, adverse event; AR, adverse reaction.

## Data Availability

The datasets presented in this article are not readily available due to Kaneka’s ownership of the intellectual property. Requests to access the datasets should be directed to the study sponsor (Kaneka, Osaka, Japan).

## Supplementary Materials

The following supporting information can be downloaded at: https://www.mdpi.com/article/10.3390/antiox15070806/s1, Figure S1: Effect of treatment on symptoms change; Figure S2: Regressions between memory and processing-time composite scores and change in CoQ10 levels, D-ROMS, and HsCRP after 90 days of supplementation with ubiquinol or placebo; Table S1: Cognitive, cardiovascular and biochemical tasks completed at various stages of the trial; Table S2: Descriptive statistics for fasting blood measures at baseline; Table S3: Estimated Marginal Means and effect of treatment on fasting blood outcomes; Table S4: Effect of treatment on risk of symptoms change; Table S5: Regressions for predicting memory composite score at trial end; Table S6: Regressions for predicting processing-time composite score at trial end. CONSORT checklist [72,73].

## Abbreviations

The following abbreviations are used in this manuscript:

CoQ10	Coenzyme Q10
MeDi	Mediterranean Diet
OM-3 PUFA	Omega-3 polyunsaturated fatty acid
CMHBS	Centre for Mental Health and Brain Science
SUT	Swinburne University of Technology
SUHREC	Swinburne University Human Research Ethics Committee
MAC-Q	Memory Complaint Questionnaire
PCA	Principal Component Analysis
RAVLT	Rey Auditory Verbal Learning Test
LM	Logical Memory
VPAT	Verbal Paired Associates Test
PRMQ	Prospective and Retrospective Memory Questionnaire
WMS-IV	Wechsler Memory Score—Fourth Edition
DSST	Digit Symbol Substitution Test
WAIS III	Wechsler Adult Intelligence Scale—Third Edition
TMT	Trail Making Test
IT	Inspection Time
PWV	Pulse Wave Velocity
HsCRP	High-Sensitivity C-Reactive Protein
D-ROMS	Diacron-Reactive Oxygen Metabolites
U.CARR	Carratelli Units
GPx	Glutathione Peroxidase
AE	Adverse Events
SAE	Serious Adverse Events
ANCOVA	Analysis of Covariance
cfPWV	Carotid–Femoral Pulse Wave Velocity
