# Supplementary material for: Effect of Ubiquinol on Cognitive Function, Blood Pressure, Arterial Stiffness, and Biomarkers of Oxidative Stress and Inflammation in the Elderly: A Randomized Trial
Source: Antioxidants (Basel). 2026 Jun 27;15(7):806. doi: 10.3390/antiox15070806 (PMC13404211; doi:10.3390/antiox15070806)
Supplement: Supplementary file 1 [file antioxidants-15-00806-s001.zip › Supplementary Data - V16 MN 25JUN2026 - Final.pdf]

Supplemental Material
Effect of Ubiquinol on Cognitive Function, Blood Pressure, Arterial Stiffness, and Biomarkers of Oxidative Stress and Inflammation in the Elderly: A Randomized Trial

1. Supplemental Methods

1.1. Testing procedures.

Assessments in this randomized, double-blind clinical trial were administered across two testing sessions, proceeded by an initial training session. The specific assessments conducted at each session are detailed in Table S1.

The selected ubiquinol dosage (200 mg/day) and intervention duration (90 days) were considered appropriate based on the evidence available at the time the study was designed. At the time, only a limited number of clinical studies had investigated the cognitive effects of CoQ10 supplementation in healthy, non-diseased populations, with our previous review of the available literature highlighting the range of dosages and intervention durations employed across human and animal studies [1]. Specifically, among studies reporting cognitive outcomes, daily doses in healthy human populations ranged from 4.5 mg to 100 mg, while studies involving clinical or disease populations utilized doses ranging from 100 mg to 2400 mg per day. More recent meta-analyses have indicated that a dose of 200 mg of CoQ10 per day and a duration of 90 days is broadly optimal for improving markers of oxidative stress [2], inflammation [3], and blood pressure [4]: factors previously shown to negatively predict cognitive function in older adults. As such, these studies provide additional, albeit retrospective, support for the appropriateness of a 200 mg daily dose.

Table S1. Cognitive, cardiovascular and biochemical tasks completed at various stages of the trial.

Study Event	Training (Day-7)	Baseline (Day 1)	Final Visit (Day 90)
Informed Consent	X		
**Cognitive Tests:**			
Logical Memory		X	X
Inspection Time	X	X	X
Verbal Paired Associates		X	X
RAVLT	X	X	X
Digit Symbol		X	X
Digit Vigilance		X	X
Trail making A and B		X	X
PRMQ		X	X
**Cardiovascular:**			
SphygmoCor	X	X	X
Blood Pressure	X	X	X
**Biochemical Assessment:**		X	X
Plasma CoQ10		X	X
D-ROMS		X	X
hsCRP		X	X
GPX		X	X
Liver enzymes		X	X

1.2. Cognitive & Mood Assessments

1.2.1. Memory Complaint Questionnaire

The Memory Complaint Questionnaire (MAC-Q) is able to quantify subjective memory complaints in prospective participants [5]. The MAC-Q consist of five questions relating to everyday memory activities (e.g., "Recalling where you have put objects (such as keys) in your home or office"). Participants respond to each question along a 5-point Likert scale indicating their current experience (e.g., 1 = "much better now", 5 = "much poorer now") relative to their experience during earlier life (i.e., high school or college). The final sixth question ("In general, how would you describe your memory compared to when you were in high school?") is weighted twice that of the initial five questions. The total range of possible scores is between 7 and 35 points.

1.2.2. Verbal Paired Associates Test I and II

The Verbal Paired Associates (VPA) test from the Wechsler Memory Scale – Fourth Edition (WMS-IV) is a validated assessment of immediate explicit verbal memory [6]. Participants committed to memorize a set of 14-word pairings presented verbally. Four of the word pairs involved related words (e.g., city-town) and ten pairs involved unrelated words (e.g., paint-big). Afterwards, participants were verbally presented with the first word of each pairing and asked to provide the corresponding word for that pairing. Each correct response was scored 1 point, and the word pair list was presented a total of four times (in a different order each time). The score range for the immediate recall phase was 0 to 56. A delayed recall phase occurred 20 to 30 minutes later, in which participants were verbally presented with the first word from each word pair, and were required to provide the corresponding word. The range of scores for the delayed recall phase was 0 to 14 points. Finally, in the delayed recognition phase, participants were verbally presented with 40 word pairs. For each word pair, participants indicated whether it was one of the word pairs they were presented with initially. The range of scores for the delayed recognition was 0 to 40 points.

1.2.3. Logical Memory I and II

The Logical Memory (LM) I and II tests from the WMS-IV (Older Adult) were administered to assess verbal memory [6]. Participants were verbally presented with two short stories (story A and story B) and were asked to immediately recall details of these stories once delivery was complete (Logical Memory I). Story A was presented twice. Scoring was based on the accurate reproduction of key story elements, with each correct response being scored 1 point. After a 30-minute delay,

participants recalled both stories again (without an initial presentation) and then completed a task assessing recognition of key story details (Logical Memory II). The range of scores for immediate recall was 0 to 53 points, delayed recall was 0 to 39 points, and delayed recognition was 0 to 23 points.

1.2.4. Digit Symbol Substitution Test

During the Digit Symbol Substitution (DSST) test, participants were presented with a grid comprising nine symbols paired to nine corresponding numerical digits (i.e., 1 to 9) [7]. A separate grid comprised several rows of numerical digits and underneath each digit was a blank space whereby participants were required to write the corresponding paired symbol. Participants were instructed to complete as much of the grid as quickly as possible over a testing period of 90 seconds. The total score was the number of digit-symbol pairs entered correctly during the 90-second testing period. Scores for the DSST could range between 0 and 90.

1.2.5. Digit Span

The Digit Span (DS) task from the Wechsler Adult Intelligence Scale – Third Edition (WAIS-III) was used to assess attention and short-term memory [8]. This test is comprised of two independent tasks, Digit Span Forward and Digit Span Backward. During the Digit Span Forward task participants were presented with two consecutive sequences of digits (1 to 9) to be immediately repeated back in the same order. If the participant correctly repeated at least one of the sequences, the sequence length was increased by 1 digit (initially there were 2 digits in the sequence with a maximum sequence length of 9). The task was terminated when a participant incorrectly recalled both sequences of the same length. The Digit Span Backward task followed the same process, though the digit sequence was repeated by the participant in the backwards or reverse order. Each correctly recited sequence was scored 1 point. The range of scores for Digit Span Forward was 0 to16; for Digit Span Backward the possible range of scores was 0 to14. The total DS score was the sum of the Digit Span Forward and Digit Span Backward scores and ranged between 0 and 30 points.

1.2.6. Trail Making Test (A and B)

The Trail Making Test (TMT) [9] consisted of two tasks requiring participants to connect circles (filled with numeric or alpha-numeric symbols) as quickly as possible. TMT-A measures processing time and attention. During TMT-A, participants were presented with circles, each containing the numbers 1 through 25. Participants were required to connect each of these circles in numerical order. TMT-B measures processing time, attention, and executive function. During TMT-B,

participants were presented with circles containing numbers (1 through to 12) and letters (A through L). Participants were required to connect these circles in ascending order, alternating between numbers and letters (i.e., 1-A-2-B-3-C-4-D etc.). Participants were required to complete both tasks without taking their pencil off the page and avoiding errors (which were to be corrected immediately before continuing). Performance was quantified by the time taken to complete the tasks.

1.2.7. Inspection Time

The Inspection Time (IT) task assessed participants' speed of information processing by determining the minimum presentation time required for accurate discrimination between two stimuli. Participants were presented (on a computer monitor) with two vertical lines of differing length joined at the top by a horizontal line. Participants were required to indicate whether they perceived the shorter vertical line as being on the left or right by pressing the corresponding response button. Each stimulus was immediately followed by a backward visual mask, which prevents further processing in iconic memory. Both the stimulus presentation time and the duration of the backward mask varied based on response accuracy, adjusting until the participant consistently achieved 80% accuracy. The minimum duration of time to achieve 80% accuracy reflected the participants processing time.

1.2.8. Rey Auditory Verbal Learning Test

During the Rey Auditory Verbal Learning Test (RAVLT) participants were presented with a fixed sequence of 15 monosyllabic words at a rate of one word every two seconds to evaluate verbal memory [10]. After each presentation, participants recalled as many words as possible, with responses recorded verbatim. This recall process was repeated five times after which a 20-minute delay occurred before completion of the delayed-recall task. In the latter task, participants recited all the words from the list of 15 words presented earlier without an initial presentation. The mean immediate recall score reflects the average number of words recited across the five immediate recall attempts. The delayed recall score reflected the number of words correctly recited after the 20-minute delay. To prevent familiarity effects, alternate word lists were used for each testing session. The range of score for mean immediate recall and delayed recall phases were 0 to 15 points.

1.2.9. The Prospective and Retrospective Memory Questionnaire

The Prospective and Retrospective Memory Questionnaire (PRMQ) assesses self-reported (i.e., subjective) memory failures in daily life [11]. The PRMQ consists of 16 items; 8 items relate to prospective memory failures (related to future intentions), and the remaining 8 items relate to retrospective memory failures (related to past events). Participants rated

each item on a 5-point scale reflecting the frequency they experience these memory failures (ranging from 1 - never, to 5 -very often). The total score, ranging from 16 to 80, reflected the overall frequency of memory failures, with higher scores indicating more frequent memory lapses. Along with the overall prospective and retrospective memory subscales, there are four additional subscales. These four subscales reflect short-term and long-term memory, as well as self-cued or environmental cued memory. Each subscale is calculated using responses from combinations of 8 survey items with the score range for each subscale being between 8 and 40 [12].

1.2.10. The Profile of Mood States

The Profile of Mood States (POMS) is a self-report measure of mood [13]. The POMS is comprised of 65 mood related adjectives (e.g., "friendly", "spiteful", "cheerful", "considerate"). Participants were required to indicate the extent to which they identified with each of these adjectives over the previous week along a 5-point-Likert scale ("not at all" to "extremely"). These items are summarized into 6 factors: 'tension-anxiety', 'confusion-bewilderment', 'anger-hostility', 'depression-dejection', 'fatigue-inertia', and 'vigor-activity'. A 'total mood disturbance' score can also be calculated by summing the first 5 factor scores and subtracting the 'vigor-activity' score. Higher scores for each factor, except for 'vigor-activity', reflect greater mood disturbance. The POMS has been shown to be a valid and reliable method of mood assessment in older adults [14].

1.3. Cardiovascular Measures

1.3.1. Brachial and Aortic Blood Pressures

Cardiovascular function involved a non-invasive assessment of brachial and aortic blood pressures using a SphygmoCor XCEL device (Model XCEL, AtCor Medical, Sydney, Australia). Following a five-minute rest period with the participant lying in the supine position, the Sphygomocor XCEL device automatically inflated and deflated a brachial cuff three times to obtain brachial blood pressures (the software automatically calculated mean systolic and diastolic pressures). Following this, the Sphygmocor XCEL device cuff automatically reinflated a fourth time to non-invasively determine the aortic pressure waveform, which was used to calculate aortic blood pressures, as well as measure reflecting peripheral arterial stiffness.

1.3.2. Central Femoral Pulse Wave Velocity (cfPWV)

Carotid-femoral pulse wave velocity (cfPWV) - the gold standard non-invasive method for assessing aortic arterial stiffness [15]

was measured using the SphygmoCor XCEL device. PWV represents the speed at which a pulse wave travels along an artery, with higher velocities indicating greater arterial stiffness. As the aorta is the primary site of arterial stiffness, cfPWV is measured between the carotid and femoral arteries. During the assessment, participants remained in a supine position while a thigh cuff is placed over the femoral artery and the distance between the carotid and femoral sites is measured. A pressure-sensitive probe was then applied to the carotid artery to capture its pressure waveform. Once a stable signal was obtained, the femoral cuff inflated, and the SphygmoCor XCEL device automatically calculated cfPWV based on the recorded data.

1.4. Biochemical Measures

1.4.1. Plasma CoQ10

Blood was collected into a 6 mL lithium heparin-coated tube, inverted and immediately wrapped in aluminum foil to protect the sample from light. The tube was centrifuged at 4000 rpm for 10 minutes at 4°C. Plasma was extracted and stored at -20°C prior to being sent to ACL for further analysis. ACL reported that analysis involved the sample being prepared by simple and direct protein precipitation with ethanol containing internal standard before being vortex mixed and centrifuged. Detection was then performed using an AB Sciex 4500 triple quadrapole mass spectrometer. The total plasma CoQ10 (as oxidized CoQ10 – ubiquinone) was then determined and reported in nmol/L.

1.4.2. High Sensitivity C-Reactive protein

Blood was collected into an 8.5 mL serum separator tube, inverted then allowed to clot for 30 minutes at room temperature before being centrifuged at 4000 rpm for 10 minutes. This sample was then sent to a commercial pathology lab (Australian Clinical Labs [ACL], Melbourne) for High sensitivity C-reactive protein (HsCRP) as well as liver enzymes (Alkaline Phosphatase, Gamma-glutamyl transferase, Aspartate Transaminase, & Alanine Transaminase) and other fasting blood measures (Sodium, Potassium, Chloride, Bicarbonate, Urea, Creatinine, eGFR, Total Protein, Albumin, Globulin, & Total Bilirubin). Serum HsCRP levels are reports in mg/L

1.4.3. Diacron Reactive Oxygen Metabolites

Blood was collected into a 6 mL lithium heparin-coated tube and subsequently inverted. The tube was then centrifuged at 2300 rpm for 10 minutes at 4°C. Plasma was extracted and stored at -80°C prior to being analyzed at the Baker HDI (Melbourne) to assess Diacron Reactive Oxygen Metabolites (D-ROMS) levels. The D-ROMS assay measures reactive oxygen metabolites derived from free radicals with unpaired electrons. These species rapidly form reactive derivatives in plasma and cells, which retain oxidative potential. When exposed to a buffered

chromogenic substrate, these derivatives form a colored compound measurable photometrically at 505 nm. The absorbance is directly proportional to the ROM concentration in the sample. Results were expressed in Carratelli Units (U.CARR), where 1 U.CARR = 0.08 mg H_2O_2/dL. Interpretation of values is as follows: 250–300 U.CARR indicates normal oxidative balance; 301–320 is borderline; 321–340 indicates mild oxidative stress; 341–400, moderate; 401–500, high; and >500 U.CARR, very high oxidative stress.

1.4.4. Glutathione Peroxidase

Blood from the same lithium heparin-coated tube used for D-ROMS was also used for glutathione peroxidase (GPx) analysis. After removal of the plasma and white buffy coat (leukocytes), the erythrocytes remaining were lysed by diluting 1:10 in ice-cold HPLC-grade water. Thereafter, the erythrocyte lysate was centrifuged at 11,400 rpm for 15 min at 4°C and stored at -80°C. GPx activity analysis utilized the GPx Assay kit (Cayman Chemicals Item # 703102) according to manufacturer's instructions. Once the reaction was initiated by addition of Cumene Hydroperoxide, the absorbance at 340nm was read every minute over a 10-minute period. The reaction rate was then calculated according to the formula: GPx Activity = $(DA_{340}/min)/0.00373 \mu M^{-1}$ x (0.19ml/0.02ml) x Sample dilution = nmol/min/ml. Higher GPx rates are reflective of greater antioxidant capacity.

1.5. Symptoms Checklist

The Symptoms Checklist was completed at the start of the in-person testing sessions (baseline & 90 days), but also by participants online after 30- and 60-days supplementation of Ubiquinol. Briefly, the Symptoms Checklist comprised of 33 items (e.g., 'abdominal pain', 'changes in sleep pattern/quality', or 'urinary frequency changes/difficulty passing urine'), with participant required to indicate yes/no if they have experienced a change in any of the listed symptoms, from their normal level of function, in the past week. The Symptoms Checklist was completed to help identify any additional changes to participant health not reported when researchers debriefed participants at the end of each visit (including online testing 'visits') to identify potential adverse events

When analyzing data from the Symptoms Checklist, we compared the proportion of participants in the Ubiquinol to the proportion of participants in the placebo group affirming a change in each item after any follow-up visit (i.e., 30, 60, or 90 days) and report the subsequent risk difference (treated minus untreated). The 95% confidence interval was computed using a standard two-proportion large sample formula. A significant difference in risk is indicated when the 95% confidence does not include '0' in the reported range.

2. Supplemental Results

2.1. Fasting Blood Outcomes

The only fasting blood measure which was significantly different between groups at baseline (when only considering participants who completed the trial) was Alkaline Phosphatase (ALP). As indicated in Table S2 below, participants in the CoQ10 treatment group, who completed the trial, had significantly lower ALP levels at baseline than participants who received the placebo.

Analyses of Covariance (ANCOVA) adjusting for age (at baseline), sex, and baseline values were performed to examine for group differences in fasting blood measures after 90 days CoQ10 or placebo supplementation. As indicated in Table S3 below, no significant group differences in any of the fasting blood measures were apparent after 90 days supplementation.

Table S2. Descriptive Statistics for Fasting Blood Measures at Baseline

Outcome	CoQ10			Placebo			*t*	*p*
	n	Mean	SD	n	Mean	SD		
Sodium (mmol/L)	54	140.02	2.07	47	139.66	2.52	0.79	0.43
Potassium (mmol/L)	54	4.44	0.39	45	4.41	0.34	0.43	0.67
Chloride (mmol/L)	54	105.43	2.43	47	104.87	2.53	1.12	0.27
Bicarbonate (mmol/L)	54	27.93	2.14	47	27.87	2.77	0.11	0.91
Urea (mmol/L)	54	6.11	2.00	47	5.86	1.55	0.69	0.49
Creatinine (µmol/L)	54	79.94	16.21	47	77.47	15.70	0.78	0.44
eGFR (mL/min/1.73m^2)	50	76.82	11.03	43	76.79	11.87	0.01	0.99
Total Protein (g/L)	54	69.15	4.18	47	68.77	3.74	0.48	0.63
Albumin (g/L)	54	40.04	2.58	47	39.60	2.72	0.84	0.41
Alkaline Phosphatase (U/L)	54	65.69	12.17	45	72.67	19.46	-2.09	0.04
Total Bilirubin (µmol/L)	54	12.83	5.09	47	13.15	8.44	-0.23	0.82
Gamm-glutamyl transferase (U/L)	54	24.43	32.89	47	23.13	14.50	0.25	0.80
Aspartate Transaminase (U/L)	54	24.96	5.98	47	25.47	6.45	-0.41	0.68
Alanine Transaminase (U/L)	53	24.49	8.56	46	22.00	8.90	1.42	0.16
Globulin (g/L)	54	29.11	3.39	47	29.17	2.41	-0.10	0.92

Note: Levene's Test of Equality of Variances was significant for *Alkaline Phosphatase* and *Globulin*, subsequently t-tests were performed with equal variances not being assumed

Table S3. Estimated Marginal Means & Effect of Treatment on Fasting Blood Outcomes

Outcome	90 Days Follow-up						Effect of Treatment				
	CoQ10			Placebo			F	p	Mean Difference at 90 Days	95% CI	η²
	n	EMM	SE	n	EMM	SE					
Sodium (mmol/L)	51	139.95	0.63	46	139.99	0.67	0.01	0.93	-0.03	-0.78, 0.72	0.00
Potassium (mmol/L)	51	4.36	0.13	44	4.34	0.14	0.05	0.83	0.02	-0.14, 0.17	0.00
Chloride (mmol/L)	51	105.29	0.71	46	105.05	0.75	0.32	0.57	0.24	-0.60, 1.08	0.00
Bicarbonate (mmol/L)	51	27.69	0.64	46	27.88	0.68	0.26	0.61	-0.19	-0.95, 0.57	0.00
Urea (mmol/L)	51	6.04	0.35	46	6.25	0.37	1.02	0.32	-0.21	-0.63, 0.21	0.01
Creatinine (µmol/L)	51	76.44	2.06	46	78.81	2.19	3.75	0.06	-2.38	-4.82, 0.06	0.04
eGFR (mL/min/1.73m^2)	46	78.37	2.18	40	76.01	2.33	2.99	0.09	2.36	-0.36, 5.07	0.04
Total Protein (g/L)	51	70.06	1.12	46	70.01	1.18	0.01	0.94	0.05	-1.27, 1.37	0.00
Albumin (g/L)	51	39.52	0.72	46	39.75	0.76	0.28	0.60	-0.23	-1.08, 0.62	0.00
Alkaline Phosphatase (U/L)	51	69.58	3.35	44	71.48	3.51	0.87	0.35	-1.90	-5.93, 2.14	0.01
Total Bilirubin (µmol/L)	51	13.04	1.22	46	12.27	1.29	1.15	0.29	0.77	-0.66, 2.21	0.01
Gamm-glutamyl transferase (U/L)	51	22.36	3.44	46	22.94	3.65	0.08	0.78	-0.58	-4.64, 3.49	0.00

Aspartate Transaminase (U/L)	51	26.47	2.45	46	26.20	2.58	0.04	0.85	0.28	-2.61, 3.16	0.00
Alanine Transaminase (U/L)	50	23.60	2.76	44	22.61	2.95	0.36	0.55	1.00	-2.32, 4.31	0.00
Globulin (g/L)	51	30.42	0.78	46	30.17	0.82	0.30	0.59	0.25	-0.67, 1.17	0.00

2.2. Symptoms Changes

The effect of Ubiquinol supplementation on symptoms changes is displayed in Table S3 and Figure S1. Overall, there appeared to be no significant difference in symptoms changes between participants who received Ubiquinol or the placebo. However, there was evidence suggesting a modest reduction in 'muscle pain' in those treated with Ubiquinol versus those who received placebo (see Table S4).

Table S4. Effect of Treatment on Risk of Symptoms Change

Symptom	Risk Difference	
Allergies	0.095	-0.046, 0.237
Coldness or Numbness	0.071	-0.081, 0.222
Decreased Stress	0.039	-0.064, 0.142
Facial Flushing	0.031	-0.091, 0.154
Bowel Changes	0.030	-0.039, 0.099
Skin Changes	0.029	-0.129, 0.187
Changes in Mood	0.028	-0.013, 0.159
Eye Issues	0.024	-0.115, 0.162
Easy Bruising/Bleeding	0.017	-0.016, 0.050
Abdominal Pain	0.013	-0.048, 0.074
Vomiting	0.000	0.000, 0.000
Mouth Issues	-0.002	-0.118, 0.113
Diarrhoea	-0.012	-0.100, 0.077
Swallowing Difficulty	-0.025	-0.090, 0.041
Shortness of Breath	-0.027	-0.157, 0.102
Muscle Weakness	-0.027	-0.157, 0.102
Dream Changes	-0.031	-0.168, 0.106
Nasal Issues	-0.034	-0.200, 0.133
Indigestion	-0.040	-0.158, 0.077
Heart Palpitations	-0.042	-0.098, 0.015
Muscle Tremors	-0.042	-0.098, 0.015
Urinary Changes	-0.053	-0.156, 0.050
Appetite Changes	-0.061	-0.184, 0.061
Constipation	-0.062	-0.131, 0.006
Nausea	-0.066	-0.151, 0.018
Energy Changes	-0.070	-0.250, 0.111
Dizzyness	-0.070	-0.168, 0.028
Digestion	-0.070	-0.168, 0.028
Sleep Changes	-0.071	-0.238, 0.095
ColdNumb	-0.086	-0.220, 0.049
Headache	-0.100	-0.275, 0.075
Increased Stress	-0.127	-0.269, 0.014
Muscle Pain	-0.148	-0.293, -0.004

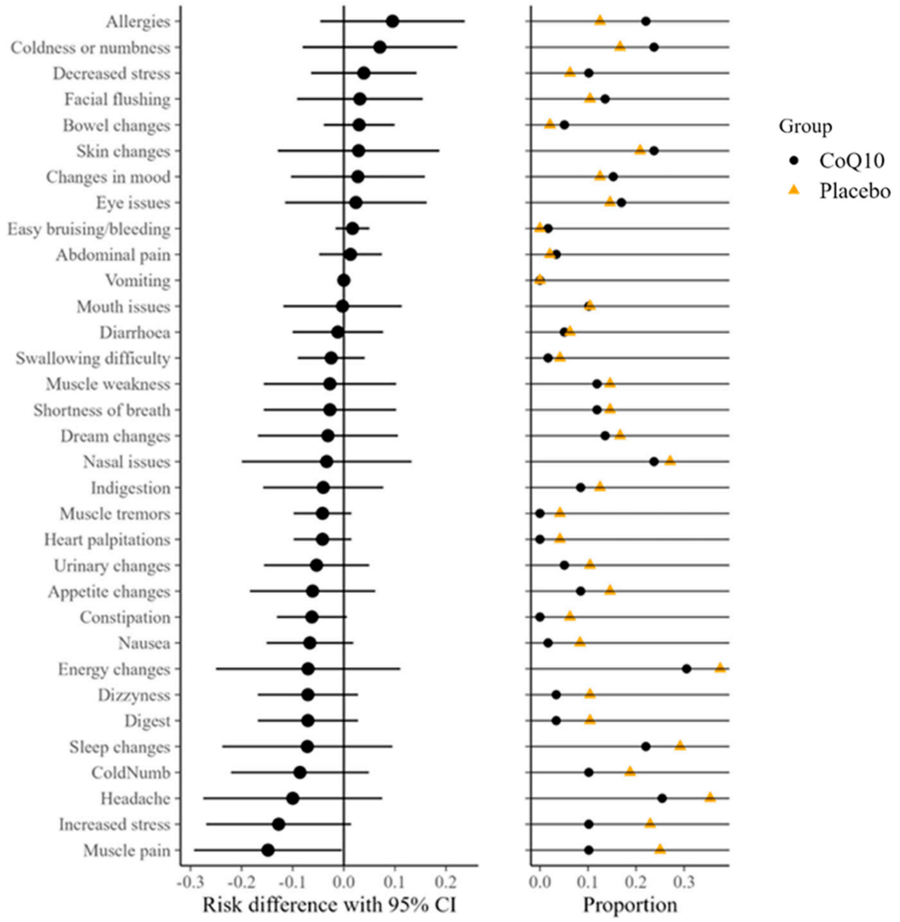

Figure S1. Effect of Treatment on Symptoms Change

2.3. Regression Analyses

Regression analyses were conducted within each treatment group to examine associations between final visit composite memory and processing time scores and corresponding biomarkers (i.e., changes in serum CoQ10, D-ROMs, and hsCRP). Regressions for Memory Composite Score and Processing Time are presented in Tables S5-S6., and all non-significant regression outcomes are presented in Figure S2.

Table S5. Regressions for Predicting Memory Composite Score at Trial End.

Predictor	Treatment	n	Unstandardized beta	p-value	95% CI
Change in CoQ10	Ubiquinol	50	0.08	0.03*	0.01, 0.16
	Placebo	43	0.04	0.84	-0.36, 0.44
Change in D-ROMS	Ubiquinol	46	-1.54	0.04*	-2.98, -0.09
	Placebo	37	0.74	0.56	-1.83, 3.31
Change in GPx	Ubiquinol	46	-0.12	0.60	-0.58, 0.34
	Placebo	39	-0.08	0.80	-0.69, 0.54

Change in HsCRP	Ubiquinol	48	0.02	0.72	-0.10, 0.15
	Placebo	41	-0.06	0.10	-0.14, 0.01

Note: Linear regression models also included age, sex, level of education, and baseline memory composite score. ***Abbreviations***: *CI*, Confidence Interval*; CoQ10,* Co-enzyme Q10; *D-ROMS*, Diacron Reactive Oxygen Metabolites; *GPx*, Glutathione Peroxidase*; HsCRP*, High Sensitivity C-reactive protein. * $p < 0.05$

Table S6. Regressions for Predicting Processing Time Composite Score at Trial End.

Predictor	Treatment	n	Unstandardized beta	p-value	95% CI
Change in CoQ10	Ubiquinol	46	-0.02	0.64	-0.11, 0.07
	Placebo	40	-0.02	0.89	-0.37, 0.32
Change in D-ROMS	Ubiquinol	44	-0.14	0.86	-1.77, 1.49
	Placebo	36	0.12	0.90	-1.94, 2.19
Change in GPx	Ubiquinol	44	-0.22	0.33	-0.67, 0.23
	Placebo	38	-0.09	0.70	-0.55, 0.38
Change in HsCRP	Ubiquinol	44	-0.12	0.07	-0.25, 0.01
	Placebo	39	0.07	0.03*	0.01, 0.13

Note: Linear regression models also included age, sex, level of education, and baseline processing time composite score. *Abbreviations*: *CI*, Confidence Interval. *CI*, Confidence Interval*; CoQ10,* Co-enzyme Q10; *D-ROMS*, Diacron Reactive Oxygen Metabolites; *GPx*, Glutathione Peroxidase*; HsCRP*, High Sensitivity C-reactive protein. * $p < 0.05$.

A.

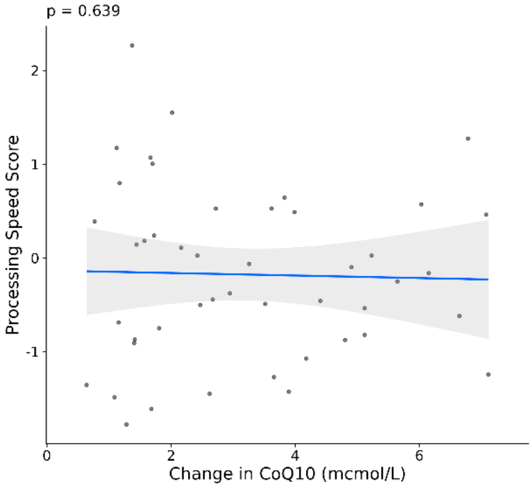

Processing Time Composite Score and Serum CoQ10 level change at 90 days for the ubiquinol group.

B.

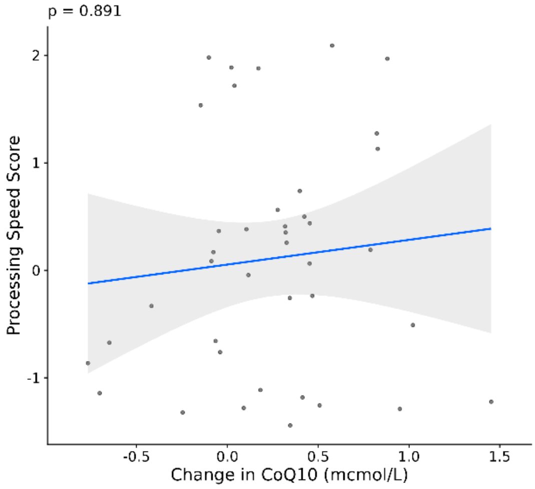

Processing Time Composite Score and Serum CoQ10 level change at 90 days for the Placebo group.

C.

D.

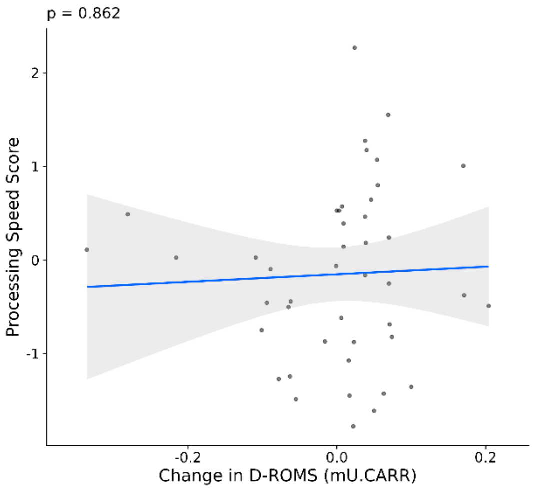

Processing Time Composite Score and DROMS level change at 90 days for the ubiquinol group.

E.

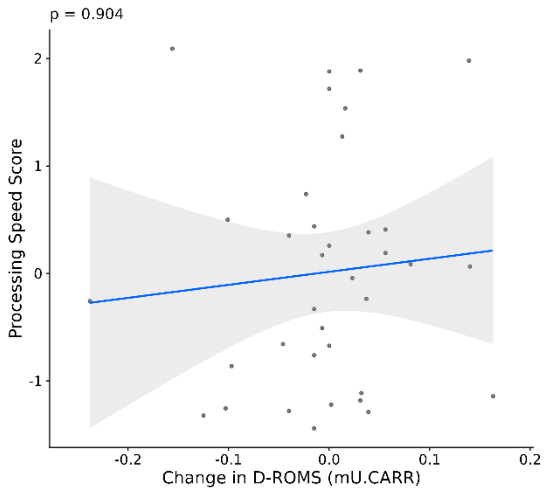

Processing Time Composite Score and DROMS level change at 90 days for the Placebo group.

F.

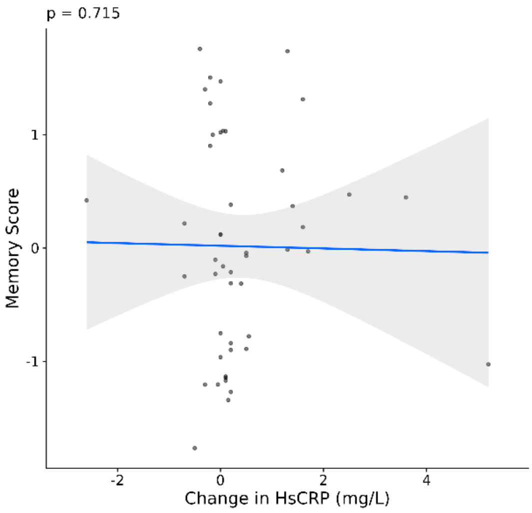

Memory Composite Score and HsCRP level change at 90 days for the ubiquinol group.

C.

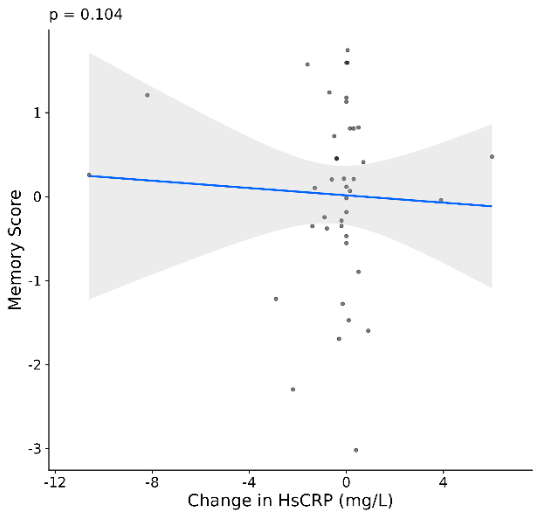

Memory Composite Score and HsCRP level change at 90 days for the Placebo group.

D.

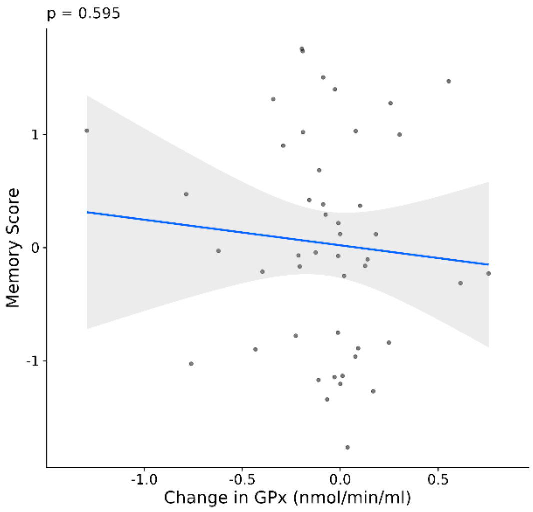

Memory Composite Score and GPX level change at 90 days for the ubiquinol group.

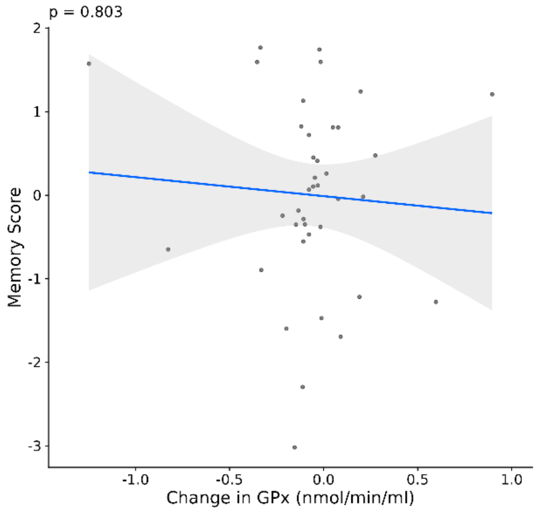

Memory Composite Score and GPX level change at 90 days for the Placebo group.

E.

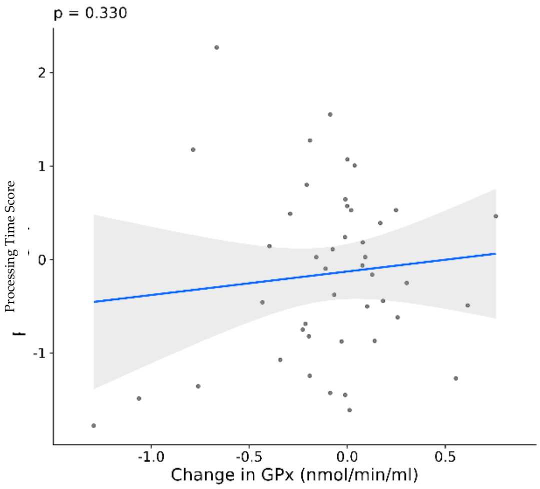

Processing Time Composite Score and GPX level change at 90 days for the ubiquinol group.

F.

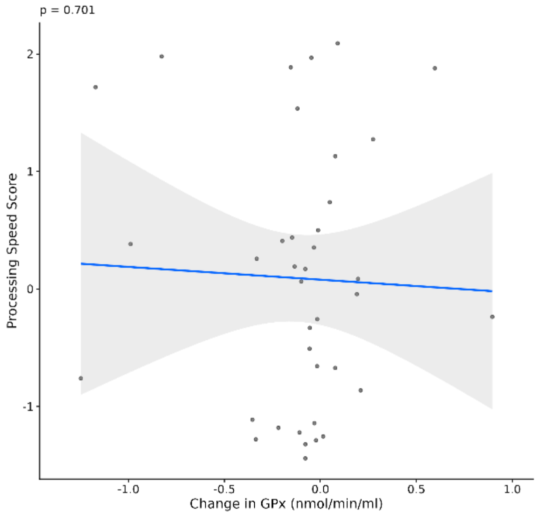

Processing Time Composite Score and GPX level change at 90 days for the Placebo group.

Figure S2. Regressions between Memory and Processing Time Composite Scores and change in CoQ10 levels, D-ROMs, hsCRP and GPX after 90 days of supplementation with ubiquinol or placebo.

References

1. Nankivell, M.C.; Rosenfeldt, F.; Pipingas, A.; Pase, M.P.; Reddan, J.M.; Stough, C. Coenzyme Q10 and Cognition: A Review. *Nutrients* **2025**, *17*, doi:10.3390/nu17172896.
2. Akbari, A.; Mobini, G.R.; Agah, S.; Morvaridzadeh, M.; Omidi, A.; Potter, E.; Fazelian, S.; Ardehali, S.H.; Daneshzad, E.; Dehghani, S. Coenzyme Q10 Supplementation and Oxidative Stress Parameters: A Systematic Review and Meta-Analysis of Clinical Trials. *Eur. J. Clin. Pharmacol.* **2020**, *76*, 1483–1499, doi:10.1007/s00228-020-02919-8.
3. Hou, S.; Tian, Z.; Zhao, D.; Liang, Y.; Dai, S.; Ji, Q.; Fan, Z.; Liu, Z.; Liu, M.; Yang, Y. Efficacy and Optimal Dose of Coenzyme Q10 Supplementation on Inflammation-Related Biomarkers: A GRADE-Assessed Systematic Review and Updated Meta-Analysis of Randomized Controlled Trials. *Mol. Nutr. Food Res.* **2023**, *67*, doi:10.1002/mnfr.202200800.
4. Karimi, M.; Pirzad, S.; Hooshmand, F.; Shirsalimi, N.; Pourfaraji, S.M.A. Effects of Coenzyme Q10 Administration on Blood Pressure and Heart Rate in Adults: A Systematic Review and Meta-Analysis of Randomized Controlled Trials. *International Journal of Cardiology: Cardiovascular Risk and Prevention* **2025**, *26*, doi:10.1016/j.ijcrp.2025.200424.
5. Crook, T.H.; Feher, E.P.; Larrabee, G.J. Assessment of Memory Complaint in Age-Associated Memory Impairment: The MAC-Q. *Int. Psychogeriatr.* **1992**, *4*, 165–176, doi:10.1017/S1041610292000991.
6. Wechsler, D. Wechsler Memory Scale--Fourth Edition (WMS-IV) 2009.
7. Ryan, J.J.; Kreiner, D.S. Clinical Applications of the Digit Symbol-Coding Subtest. In *The quantified process approach to neuropsychological assessment*; A. M. Poreh, Ed.; Taylor & Francis, 2006; pp. 53–82.
8. Wechsler, D. WAIS-III: Administration and Scoring Manual 1997.
9. Bowie, C.R.; Harvey, P.D. Administration and Interpretation of the Trail Making Test. *Nat. Protoc.* **2006**, *1*, 2277–2281, doi:10.1038/nprot.2006.390.
10. Rey, A. Rey Auditory Verbal Learning Test (RAVLT) 1958.
11. Smith, G.; Della Sala, S.; Logie, R.H.; Maylor, E.A. Prospective and Retrospective Memory in Normal Ageing and Dementia: A Questionnaire Study. *Memory* **2000**, *8*, 311–321, doi:10.1080/09658210050117735.
12. Crawford, J.R.; Henry, J.D.; Ward, A.L.; Blake, J. The Prospective and Retrospective Memory Questionnaire (PRMQ): Latent Structure, Normative Data and Discrepancy Analysis for Proxy-Ratings. *Br. J. Clin. Psychol.* **2006**, *45, 83–104*, https://doi:10.1348/014466505X28748
13. McNair, D.M.; Lorr, M.; Droppleman, L.F. Manual for the Profile of Mood States. *Educational and Industrial Testing Service* **1971**.
14. Gibson, S.J. The Measurement of Mood States in Older Adults. *J Gerontol B Psychol Sci Soc Sci* **1997**; *52, 167-174.*
15. Brunner-La Rocca, H.P. Towards Applicability of Measures of Arterial Stiffness in Clinical Routine. *Eur. Heart J.* **2010**, *31*, 2320–2322, doi:10.1093/eurheartj/ehq211.
